# Cell Specific Changes of Autophagy in a Mouse Model of Contusive Spinal Cord Injury

**DOI:** 10.3389/fncel.2018.00164

**Published:** 2018-06-12

**Authors:** Teresa Muñoz-Galdeano, David Reigada, Ángela del Águila, Irene Velez, Marcos J. Caballero-López, Rodrigo M. Maza, Manuel Nieto-Díaz

**Affiliations:** ^1^Laboratory of Molecular Neuroprotection, UDI-HNP, Hospital Nacional de Parapléjicos (SESCAM), Toledo, Spain; ^2^Division of Developmental Biology, Cincinnati Children's Hospital Medical Center, Cincinnati, OH, United States

**Keywords:** neural cell, LC3, Beclin 1, autophagic flux, cell death

## Abstract

Autophagy is an essential process of cellular waist clearance that becomes altered following spinal cord injury (SCI). Details on these changes, including timing after injury, underlying mechanisms, and affected cells, remain controversial. Here we present a characterization of autophagy in the mice spinal cord before and after a contusive SCI. In the undamaged spinal cord, analysis of LC3 and Beclin 1 autophagic markers reveals important differences in basal autophagy between neurons, oligodendrocytes, and astrocytes and even within cell populations. Following moderate contusion, western blot analyses of LC3 indicates that autophagy increases to a maximum at 7 days post injury (dpi), whereas unaltered Beclin 1 expression and increase of p62 suggests a possible blockage of autophagosome clearance. Immunofluorescence analyses of LC3 and Beclin 1 provide additional details that reveal a complex, cell-specific scenario. Autophagy is first activated (1 dpi) in the severed axons, followed by a later (7 dpi) accumulation of phagophores and/or autophagosomes in the neuronal soma without signs of increased initiation. Oligodendrocytes and reactive astrocytes also accumulate phagophores and autophagosomes at 7 dpi, but whereas the accumulation in astrocytes is associated with an increased autophagy initiation, it seems to result from a blockage of the autophagic flux in oligodendrocytes. Comparison with previous studies highlights the complex and heterogeneous autophagic responses induced by the SCI, leading in many cases to contradictory results and interpretations. Future studies should consider this complexity in the design of therapeutic interventions based on the modulation of autophagy to treat SCI.

## Introduction

Trauma to the spinal cord triggers a number of pathophysiological processes that deeply alter its tightly regulated cellular environment and contributes to the resulting functional deficits (Profyris et al., 2004). Recent studies reveal that macroautophagy is among the cellular processes altered after SCI (Kanno et al., 2009b; Rodríguez-Muela and Boya, 2012; Lipinski et al., 2015; Nikoletopoulou et al., 2015). Macroautophagy (hereafter referred to as autophagy) is a highly conserved lysosomal degradative process responsible for the turnover of long-lived proteins and the removal of cellular waste like aberrant proteins, lipid droplets and dysfunctional cytoplasmic organelles (Mizushima et al., 2008). Autophagy is a dynamic process -termed autophagic flux- which comprises the initiation and formation of autophagic vesicles (phagophores and autophagosomes), the engulfing of cellular waste by these vesicles, and finally the fusion of the loaded autophagosomes with lysosomes (autolysosomes) to degrade their cargoes (Klionsky, 2000; Mizushima et al., 2011). Well known as a cellular response to noxious stimuli such as starvation (Levine and Klionsky, 2004; Mizushima and Komatsu, 2011), autophagy is in fact constitutively active in all cells to maintain their homeostasis –being critical in terminally differentiated cells such as neurons and oligodendrocytes—(Komatsu et al., 2006; Smith et al., 2013). Impairment of the autophagic flux causes the accumulation of damaged organelles and protein aggregates that interfere with cellular function and may lead to cell death processes as observed in neurodegenerative diseases such as Alzheimer's, Huntington's and Parkinson's diseases (Shintani and Klionsky, 2004; Levine and Kroemer, 2008; Mizushima et al., 2008; Choi et al., 2013; Nixon, 2013), or in lysosomal storage diseases (Settembre et al., 2008a,b; Lieberman et al., 2012).

Previous studies in different murine models of SCI have revealed important alterations in the autophagic process (Lipinski et al., 2015). However, the precise nature of these alterations remains controversial. Some authors have described significant increases in autophagosome abundance during the first 24 h after SCI based on the expression levels of the autophagosome marker MAP1LC3B-II (hereafter referred to as LC3-II) (Chen et al., 2012; Hao et al., 2013; Hou et al., 2014; Liu et al., 2015), whereas others have only detected a delayed increase of this marker 3 to 7 days after SCI (Kanno et al., 2011; Sekiguchi et al., 2012; Zhang et al., 2013a, 2014; Berliocchi et al., 2015). It also remains controversial whether the rise in autophagosome abundance is due to the initiation of the autophagic process or to a blockage in the latest stages of the autophagic flux. In this sense, some authors have described that the expression of Beclin 1, a key-protein in the initiation of autophagy (He and Levine, 2010), increases after SCI, supporting the first hypothesis, an increase of autophagy initiation (Kanno et al., 2011; Sekiguchi et al., 2012; Wang et al., 2014; Berliocchi et al., 2015). Other works, however, have reported unaltered levels of Beclin 1 and other autophagy initiation proteins (Sarkar et al., 2014; Liu et al., 2015), which would support the alternative hypothesis, an impairment in the degradative phase of the flux. Disagreements extend to the functional consequences of the altered autophagy, which can either lead to cell death (Walker et al., 2012; Zhang et al., 2013b; Yin et al., 2014; Wang et al., 2015) or protect neural cells following SCI (Sekiguchi et al., 2012; Chen et al., 2013; Tang et al., 2014; Gao et al., 2015; Goldshmit et al., 2015; Zhou et al., 2015). Determining how the process changes after the trauma and which are consequences of these changes is key to establish autophagy as a therapeutic target for SCI treatment.

The present study stems from this idea, aiming to determine how autophagy changes in a mice model of contusive SCI, and to establish the spatiotemporal and cellular distribution of these changes. Our results reveal that contusive SCI in mice induces a general increase of autophagosome abundance during the sub-acute phase (7 days after injury). A detailed evaluation of autophagy in the main spinal cord neural cells (neurons, oligodendrocytes, and astrocytes) shows that autophagic responses vary depending on cell type and location relative to injury epicenter.

## Material and methods

### Animals

All experimental procedures were carried out on wild type (Harland Laboratories, Indianapolis, USA) and GFP-LC3 transgenic mice (RIKEN Bioresource Center, Tsukuba, Japan) both with a C57BL/6 background (Mizushima et al., 2004). GFP-LC3 mice were kindly gifted by Dr. Patricia Boya (Centro de Investigaciones Biológicas, CSIC; Madrid, Spain). Mice were housed in plastic cages in a temperature and humidity controlled room, and maintained on a 12:12 h reverse light/dark cycle with free access to food and water. Animal experimental procedures were in accordance with the European Communities Council Directive 2010/63/EU, Spanish Royal Decree 53/2013 (experimental animal use regulation) and ECC Order 566/2015 (regulation of personnel formation in animal experimentation), and were approved by the Hospital Nacional de Parapléjicos Animal Care and Use Committee (ref# 63/2010). All efforts were made to minimize the number of animals used and their suffering.

### SCI procedure

Female mice of approximately 20 g of weight (12–14 weeks old) were anesthetized through isoflurane [Baxter HealthCare Corporation, MDC-10019-360-60)] inhalation (2% in oxygen for induction and 1,5% during surgery). The spinal cord T8 segment was exposed by laminectomy in the 9th thoracic vertebra (T9) and subsequently received a 50 Kdyne (moderate) contusion using an IH Spinal Cord Impactor device (Precision System & Instrumentation, Lexington, KY, USA). After surgery, animals were maintained by daily manual bladder expression for 2 weeks and by administration of analgesic buprenorfine (0.03 mg/Kg) (Reckitt Benckiser Pharmaceuticals Limited, 961425), and antibiotic enrofloxazine (0.4 mg/Kg) (Bayer AG, CN 570216.2) for 2 days. In order to confirm the degree of injury, locomotor impairment was evaluated using the Basso Mouse Scale for locomotion (BMS) (Basso et al., 2006) 2 days after injury or before euthanasia for the studies during acute phase. Animals with BMS scores above 3 in the first 2 days after injury were excluded from the study.

### Immunoblotting

At defined times after injury, animals were euthanized by administration of a lethal dosage of 50 mg/kg sodium pentobarbital (Vetoquinol, QN51AA01). A fragment of 1 cm of spinal cord centered in the injured area was sampled and homogenized in RIPA lysis buffer (Sigma, R0278) supplemented with a protease inhibitor cocktail (Roche, 11873580001), incubated for 30 min at 4°C and cleared by centrifugation (10.000 × g for 15 min at 4°C). Protein concentration of the lysates was quantified using the bicinchoninic acid method (ThermoFisher Scientific, 23227) following manufacturer's protocol. Cell lysates containing 20 μg of total protein were mixed with Laemmli buffer [2-mercaptoethanol, 0.1% (Sigma, M7154); bromophenol blue, 0.0005% (UBS Affimetrix, 12370); Glycerol, 10%; Sodium dodecyl sulfate (SDS), 2% and Tris-HCl, 63 mM (pH 6.8)] and boiled for 5 min at 100°C. After SDS-polyacrylamide gel electrophoresis (SDS-PAGE), proteins were transferred to polyvinylidene difluoride membranes (Merk Millipore, IPSN07852). Then, membranes were blocked with 5% non-fat milk or bovine serum albumin (Sigma Aldrich, G7893) diluted in Tris buffer saline plus (Fischer Scientific, BP2471), 0.05% (v/v) Tween20 (Sigma, P9416) (TBS-T) and incubated overnight at 4°C with the appropriate specific antibodies diluted in its blocking solution type at (see Table 1). Afterwards, blots were incubated at RT for 90 min with the correspondent horseradish peroxidase (HRP)-conjugated secondary antibody (see Table 1) diluted in non-fat milk blocking solution. Detection by enhanced chemiluminiscence (ECL) was performed using SuperSignal West Pico chemiluminescent assay (Thermo Fisher Scientific, 34577) according to the manufacturer's instructions. Blot images were acquired using ImageScanner III and LabScan v6.0 software (GE Healthcare Bio-Sciences AB, Uppsala, Sweden) and bands intensity was measured using ImageJ software version v1.49b (National Institutes of Health, NIH, Bethesda, MD, USA). For LC3 protein detection, transference was carried out overnight at 4°C with constant current intensity of 1.72 mA/cm^2^ followed by incubation with its specific antibody for 2 h at RT. All employed antibodies recognized the specific band or bands of expected molecular weight for their target/s without detection of any non-specific bands. Following Liu and colleagues (Liu and Xu, 2006), we used β-tubulin as a loading control to avoid artifacts associated with expression changes in other common controls.

**Table 1:** Antibodies.

**Immunoblotting antibodies**	**Comercial reference and antibody registry ID**
Anti-microtubule-associated protein 1 light chain 3 isoform B (LC3B)	Sigma-Aldrich Cat# L7543, RRID:AB_796155
Anti-tubulin β-III	Sigma-Aldrich Cat# T5293, RRID:AB_477580
Anti-p62/SQSTM1	Sigma-Aldrich Cat# P0067, RRID:AB_1841064
Anti-Beclin1 (D40C5)	Cell Signaling Technology Cat# 3495S, RRID:AB_1903911
Anti-Beclin1	Abcam Cat# ab16998, RRID:AB_725664
**Immunofluorescence antibodies**	**Comercial reference and antibody registry ID**
Anti-LC3B	MBL International Cat# PM036, RRID:AB_2274121
Anti-Beclin 1	Abcam Cat# ab16998, RRID:AB_725664
Anti-Glial Fibrillary Acidic Protein (GFAP)	Abcam Cat# ab4674, RRID:AB_304558
Anti-Neuronal Nuclei protein clon A60 (NeuN)	Merck Cat# MAB377, RRID:AB_11210778
Anti-Green Fluorescence Protein (GFP)	Roche Cat# 11814460001, RRID:AB_390913
Anti-Adenomatous Polyposis Coli (Ab-7) (APC)	Millipore Cat# OP80, RRID:AB_2057371
Anti-Beclin 1	Novus Cat# NB110-87318, RRID:AB_1201249
Pan-Axonal Neurofilament Marker SMI-312	Covance Research Products Inc Cat# SMI-312R-100, RRID:AB_509993
**Immunoblotting secondary antibodies**	**Comercial reference and antibody registry ID**
HRP-conjugated goat anti-rabbit	Thermo Fisher Scientific Cat# 31460, RRID:AB_228341
HRP-conjugated goat anti-mouse	Thermo Fisher Scientific Cat# 31430, RRID:AB_228307
**IMMUNOFLUORESCENCE SECONDARY ANTIBODIES**	
Alexa Fluor 488 goat anti-rabbit	Life Technologies Cat# A11034, RRID:AB_10562715
Alexa Fluor 488 goat anti-mouse highly-cross adsorbed	Molecular Probes Cat# A11029, RRID:AB_138404
Alexa Fluor 594 goat anti-mouse	Molecular Probes Cat# A11005, RRID:AB_141372
Alexa Fluor 594 goat anti-mouse IgG_2b_	Life Technologies Cat# A21145, RRID:AB_10374722
Alexa Fluor 594 goat anti-chicken	Molecular Probes Cat# A11042, RRID:AB_142803

### Immunofluorescence

At defined times after injury, animals were anesthetized by intraperitoneal injection of 50 mg/kg sodium pentobarbital (Vetoquinol, QN51AA01). Mice were then transcardially perfused with saline, followed by 4% paraformaldehyde (Sigma Aldrich, P6148) in 0.1 M phosphate buffer, PB, pH 7.4. 1 cm long spinal cord samples centered in the lesion epicenter were collected, post-fixed for 48 h in 4% paraformaldehyde (4°C, overnight) and cryoprotected for 2–3 days in 30% sucrose in PB (w/v). Afterwards, spinal cord samples were embedded in optimum cutting temperature compound (Sakura Finetek, 4583) and frozen at −80°C. Embedded tissue was cut in 20 μm transversal sections using a cryostat (HM560, Microm, GmbH, Walldorf, Germany) and mounted in microscope glass slides (Thermo Fisher Scientific, 1014356190). Serial sections in each slide were separated 300 μm covering the injured and the adjacent rostral and caudal segments.

To carry out the immunofluorescence staining, sections were first heated at 37°C for 45 min, rehydrated in phosphate-buffered saline, PBS (Thermo Fisher Scientific, BP399), and blocked and permeabilized by incubation for 2 h at RT in a solution composed by 5% (v/v) normal goat serum (Sigma Aldrich, G9023) and 0.2% (v/v) Triton X-100 (Sigma Aldrich, T8787) in PBS. Afterwards, sections were incubated overnight at 4°C with specific primary antibodies diluted at appropriate concentrations (see Table 1) in blocking solution, rinsed in PBS, and incubated for 2 h at RT with the appropriate Alexa Fluor-conjugated secondary antibodies (see Table 1) also diluted in blocking solution. The stained sections were mounted with a Fluorescent Mounting Medium (Agilent, S302380-2) containing the fluorescent marker of nucleic acids 4′,6-diamino-2-fenilindol, DAPI, 1.5 μg/ml (Sigma-Aldrich, D9542001). For LC3 immunostaining we followed the protocol described by Tanida et al. (Tanida et al., 2008) with some modifications. Briefly, sections were thawed and dried for 45 min at 37°C and rehydrated in TBS for 15 min at RT. Afterwards, slices were permeabilized with digitonin solution (50 μg/ml in PBS; Sigma Aldrich, D141) for 15 min at 37°C. Excess digitonin autofluorescence was quenched by incubation in ammonium chloride solution (50 mM in PBS, Sigma Aldrich, A0171) for 15 min at 37°C. Then, slices were blocked in a solution of 2% bovine serum albumin, BSA (Sigma Aldrich, A7906), 5% goat serum, 20 mM Tris-HCl pH 7.5 (UBS Affimetrics, 22637), and 150 mM NaCl (Sigma Aldrich, 31434-M) in PBS for 2 h at RT. After three washes for 10 min with TBS, sections were incubated in blocking solution with rabbit anti-LC3 primary antibody (see Table 1) at 4°C overnight. After three additional washes in TBS, sections were incubated for 2 h at RT with the appropriate Alexa fluor-conjugated secondary antibodies (see Table 1) in blocking solution. Finally, sections were washed three times in TBS and mounted with mounting medium (DAKO, S3023) supplemented with DAPI.

### Image analysis

Stained sections were examined using a High Speed Resonant Confocal Scanner Microscope or TCS SP5 Confocal Microscopy (Leica Microsystem CMS GmbH, Wetzlar, Germany) equipped with HCX PL APO CS 20x dry uv, HCX PL APO CS 40x dry uv and HCX PL APO CS 63x oil UV objectives. 20X and 40X images were captured as z-stacks using Leica Application Suite Advanced Fluorescence (LAS-AF) imaging system version 2.3 (Leica Microsystem CMS GmbH, Wetzlar, Germany). Conditions for image capturing were kept constant for all sections in each experiment. Images were processed using Adobe Photoshop CS3 software extended version 10.0.1 (Adobe Systems Incorporated, USA) and ImageJ software version v1.49b (National Institutes of Health, NIH, Bethesda, MD, USA) for quantification of cellular expression of target protein. To analyse expression in oligodendrocytes and neurons (Figures S2, S3, respectively), we first employed a local optimized threshold, after using enhancement tools from ImageJ (median filter, substract background) in 3 μm thick optical sections, to mask the cells according to specific cell type markers (NeuN for neurons, and APC for oligodendrocytes). Afterwards, we employed the cell masks in the resulting binary image to quantify LC3 or Beclin 1 signal intensity in their specific channels. We subtracted the mean signal intensity from negative controls (lacking primary antibodies) in the same slide to control for differences in background staining. Because cell body cannot be easily outlined in astrocytes, instead of analyzing individual cells we measured LC3 and Beclin-1 signal in pixels with positive signal for the astrocyte marker GFAP. We defined positive pixels as those with GFAP signal intensity over 50 in a 0 to 255 intensity scale, well above the values of negative controls. Considering that GFAP expression increases with astrocyte reactivity (Sofroniew and Vinters, 2010), we further split the resulting LC3 and Beclin-1 data between pixels with low (50–100) and high (intensity >100) GFAP signals as estimations of the signal in non-reactive and reactive astrocytes, respectively.

### Data analysis

Statistical analyses were conducted using GraphPad Prism (GraphPad Software, La Jolla, CA, USA) and R software (R Development Core Team, 2011. Vienna). One-way analysis of variance ANOVA followed by Tukey or Dunnet's Multiple Comparison post hoc tests, and Student's *t-*test were used depending to the characteristics of the data. Differences were considered significant when *p*-value < 0.05. LC3 and Beclin-1 immunofluorescence signal were represented using standard Box and Whisker plots, that is, the bottom and top of the box indicate the first and third quartiles, and the line inside corresponds to the median. The ends of the whiskers indicate the lowest and highest data within 1.5 IQR of the lower and upper quartile, therefore data outside can be considered outliers.

## Results

### Expression and distribution of autophagic protein LC3 in the naïve spinal cord

**Figure 1:**
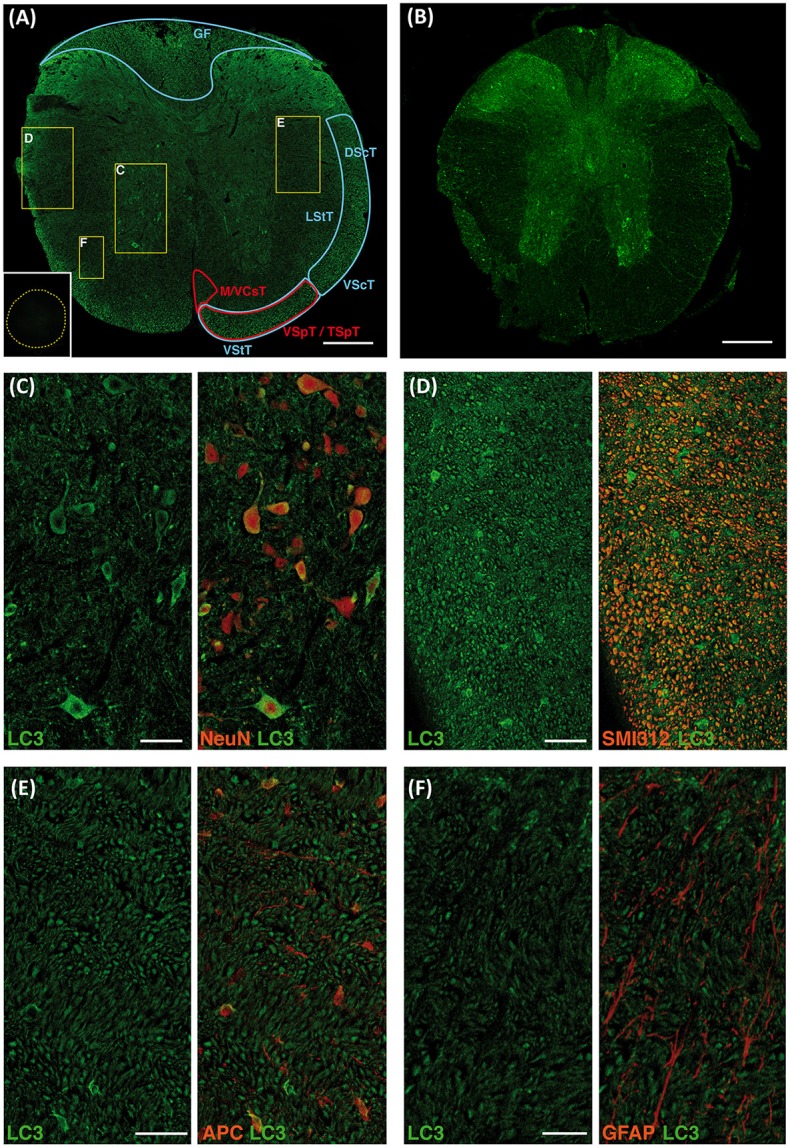
LC3 expression in neural cells of the undamage spinal cord. Representative immunofluorescence (IF) images of coronal sections of non-injured spinal cords from wild-type **(A)** and GFP-LC3 transgenic mice **(B)** showing LC3 expression in both ascending (blue areas) and descending tracts (red areas) (*n* = 3 wild type mice; *n* = 2 transgenic mice). Inset in **(A)** shows representative negative control in absence of anti-LC3 antibody where yellow dotted trace delimits the spinal cord. **(C–F)** High magnified confocal images of the different areas indicated in **(A)** showing LC3 expression (left) and co-expression with cellular markers (right) for neurons (NeuN) **(C)**, axons (SMI 312) **(D)**, oligodendrocytes (APC) **(E)**, and astrocytes (GFAP) **(F)**. Abbreviations in **(A)** correspond to: GF, gracile fasciculus; DScT, dorsal spinocerebelar tract; VScT, ventral spinocerebelar tract; LStT, lateral spinothalamic tract; VStT, ventral spinothalamic tract; MCsT, medial corticospinal tract; VCsT, ventral corticospinal tract; VSpT, vestibulospinal tract; TSpT, tectospinal tract. Scale bars correspond to 250 μm in **(A**,**B)** to 50 μm in **(C–E)** and to 25 μm in **(F)**.

Immunoblotting demonstrated that autophagic marker LC3 protein is expressed in the C57BL/6 mice spinal cord (see Figure S1). Therefore, we labeled LC3 in T9 segment sections from mice spinal cords to characterize the anatomical and cellular distribution of phagophores, autophagosomes and derived autophagic vesicles (*i.e*. autophagic structures). Although available antibodies against LC3 stain both LC3-I and LC3-II, Sarkar et al. (Sarkar et al., 2014), Castillo et al. (Castillo et al., 2013) and Matus et al. (Matus et al., 2014) demonstrated that intensity of LC3 staining in cells can be related to their actual abundance of autophagic structures. LC3 labeling was observed in different regions and neural cell types both in the white and gray matters (Figure 1A). In the white matter, co-staining with the pan-axonal neurofilament marker (anti-SMI-312) revealed the expression of LC3 in various tracts (Figure 1D), namely the ascending proprioceptive *fasciculus gracilis*, the anterior and posterior spinocerebellar tracts, the lateral and anterior spinothalamic tracts, the descending medial and anterior corticospinal tracts, and the vestibulo/tectospinal tracts (Figure 1A). Co-staining with the oligodendrocyte marker (anti-APC) showed that LC3 was also expressed in these cells, although labeling was highly heterogeneous, from strongly stained cells to others lacking appreciable signal (Figure 1E). On the other hand, co-staining with the astrocyte marker anti-GFAP revealed very few LC3 positive astrocytes restricted to the *glia limitans superficialis* (Figure 1F). In the gray matter, neuronal soma stained with anti-NeuN showed a variable LC3 immunoreactivity (Figure 1C). Thus, LC3 staining was very intense in some large neurons from the ventral horn (Redex's VII, VIII, and IX laminae), but was absent in nearby ones (See Figure 1C). LC3 immunoreactivity was also observed in the neuropil, particularly in the first three Redex's laminae at the dorsal horns. A similar LC3 staining pattern was observed in tissue samples from GFP-LC3 transgenic mice (Figure 1B).

### LC3 expression suggests that autophagosome abundance increases after spinal cord injury

**Figure 2:**
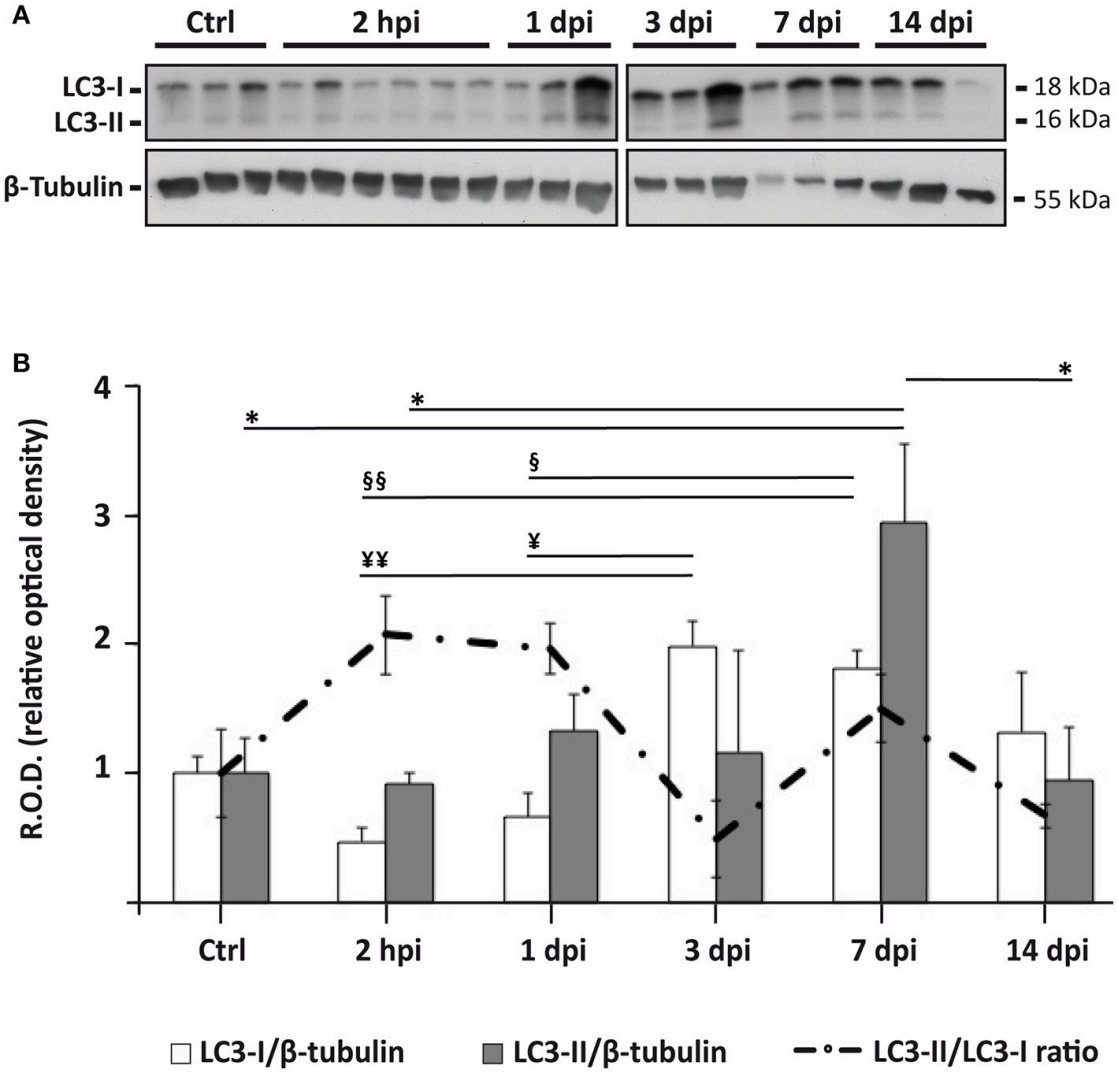
Autophagosomes accumulate 7 days after spinal cord injury. **(A)** Representative LC3 immunoblot of spinal cord lysates from control and injured animals sampled at different times post-injury (*n* = 3 per time, except *n* = 6 for 2 hpi; each lane represents an individual animal). **(B)** Densitometry of LC3 immunoblots reveals significant changes in the expression of LC3-I (white bars) and LC3-II (gray bars), and in the ratio LC3-II/LC3-I (dashed line) following SCI. Densitometry was carried out in a immunoblot different from those shown in **(A)**, in which samples were randomly distributed to avoid spatial biases (available as Figure S6). Values of LC3-I and LC3-II were both normalized to the loading control (β-tubulin) and to the mean density levels of non-injured controls. Bars represent mean ± S.E.M; *^, ¥, §^ indicate *p* < 0.05; ^¥¥, §§^ *p* < 0.01. Probabilities were estimated from one-way ANOVA followed by Tukey's *post-hoc* test.

Immunoblotting analysis of spinal cord homogenates sampled at 2 h post injury (hpi), and at 1, 3, 7, and 14 days post injury (dpi; Figure 2A) revealed that SCI caused a significant increase in LC3-II expression at 7 dpi (one-way ANOVA, *F*_(5, 17)_ = 3.554; *p* < 0.05; *p* < 0.05 respect to control, 2 hpi and 14 dpi, Tukey's post-hoc test; Figure 2B). Expression of LC3-II precursor (LC3-I) also changed significantly after injury (*F*_(5, 17)_ = 7.109; *p* < 0.001) but following a different profile (Figure 2B). LC3-I levels decreased during the first 24 h after SCI, increasing thereafter to reach a sustained high level from 3 dpi to 7 dpi (*p* < 0.05, 2 hpi and 1 dpi vs. 3 and 7 dpi; Tukey's post-hoc test). LC3-II/LC3-I ratio (an estimate of the conversion rate of LC3-I into LC3-II) augmented in the first 24 h after SCI but suddenly decreased at 3 dpi to rise again at 7 dpi, without reaching the levels observed 2 hpi or 1 dpi (Figure 2B). In summary, immunoblot results suggest that the abundance of autophagic structures remains relatively constant during the first two weeks after injury except for a sudden increase at 7 dpi. Interestingly, the behavior of LC3-II does not reflect the expression changes of its precursor LC3-I.

### LC3 shows time, spatial and cell type dependent changes after SCI

**Figure 3:**
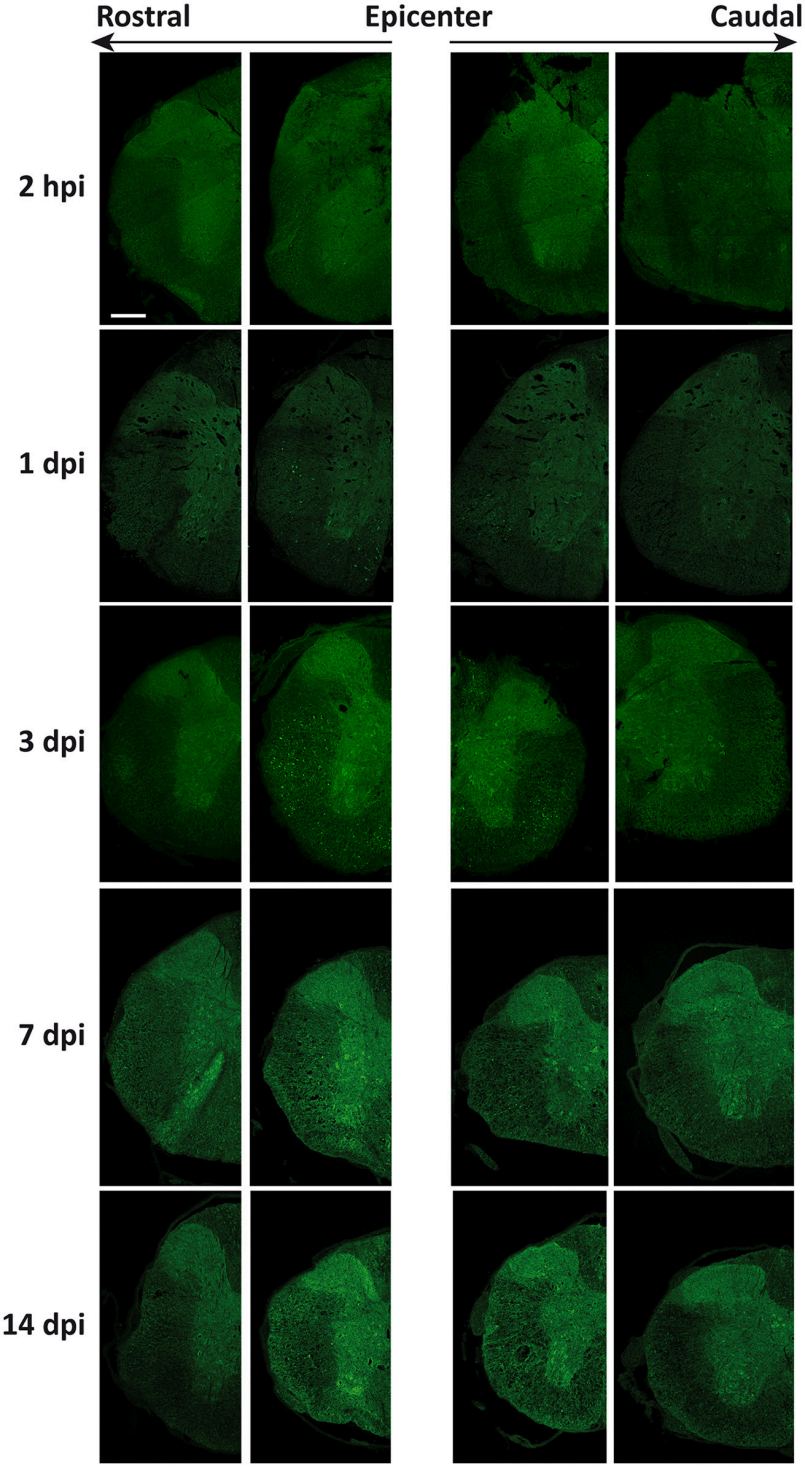
Spatio-temporal distribution of LC3 expression in the injured spinal cord. Representative immunofluorescence images of coronal sections of spinal cords from wild-type mice labeled with anti-LC3 antibody (*n* = 3 individuals per time). Sections illustrate LC3 expression at increasing times after injury **(Top to Bottom)** and at different distances from injury epicenter at both rostral **(Left)** and caudal **(Right)** directions. Farthest sections are separated about 3 mm and nearest ones about 0.5 to 1 mm from injury epicenter. Scale bar: 500 μm.

**Figure 4:**
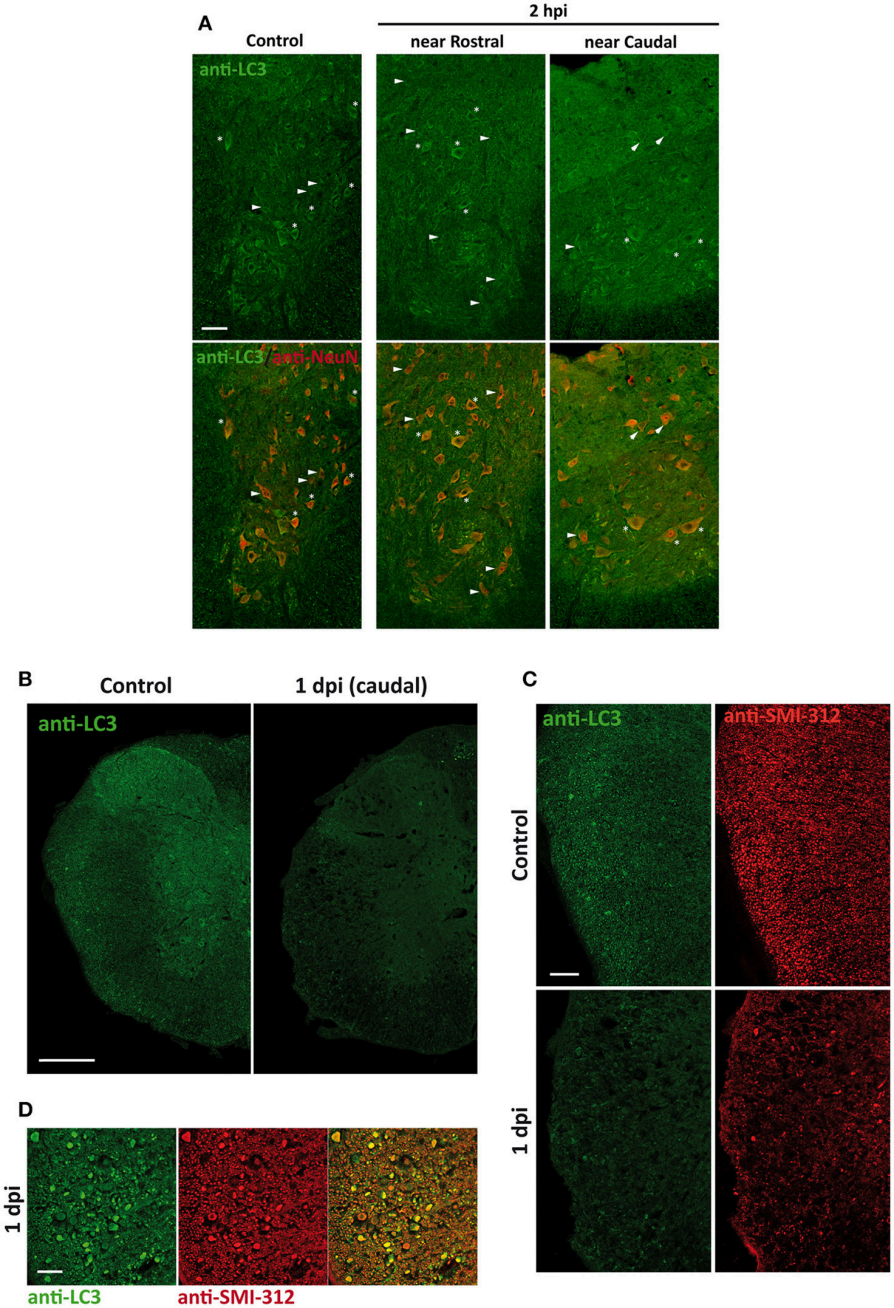
**(A)** LC3 expression increases slightly in neurons 2 h after injury (2 hpi). Representative confocal images of sections from non-injured spinal cords (left) and from spinal cords sampled 2 hpi (located 0.5–1 mm away from injury epicenter in rostral (middle) or caudal (right) direction) (*n* = 3 per condition). Sections were labeled with anti-LC3 and anti-NeuN antibodies. Merged images (bottom panels) show the co-expression of LC3 protein and neuronal marker NeuN. Heterogeneity in LC3 signal among neurons is illustrated by identifying LC3 positive (stars) and negative (arrowheads) cells. **(B)** Overall LC3 expression decreases 1 day after injury (1 dpi) but increases in axon retraction bulbs. Representative confocal images of coronal sections of non-injured (left) and 1 dpi (right) wild-type mouse spinal cords labeled with anti-LC3 antibody (*n* = 3 per condition). **(C)** Detail of the lateral white matter from control (upper panels) and 1 dpi spinal cord sections (lower panels) labeled with anti-LC3 (left) and anti-SMI-312 (axonal marker) (right). **(D)** High magnification confocal image showing LC3 (left) and SMI-312 labeling (middle) and their co-expression (right) in retraction bulbs of the lateral white matter from rostral sections 0.5–1 mm away the epicenter. Scale bars correspond to 50 μm in **(A)**, 500 μm in **(B)**, 150 μm in **(C)** and 50 μm in **(D)**.

Changes in the spatial, anatomical, and cellular distribution of LC3 were characterized by immunofluorescence in 2 hpi, 1, 3, 7, and 14 dpi spinal cord sections from wild-type mice (Figure 3). Examination of LC3 staining in the injury epicenter sections was prevented due to strong tissue autofluorescence. Confocal analysis revealed that 2 h after injury (Figure 4), LC3 signal remained similar to that observed in non-injured mice (see Figure 1). Minor differences included a slight increase in LC3 staining among neurons from the regions in direct contact with the damaged tissue (Figure 4). One day after injury, LC3 signal diminished (Figure 4B), particularly in those sections 0.5–1 mm around the injury. In the white matter, widespread loss of tracts caused a reduction of LC3 signal, mainly in caudal sections (Figure 4C). Interestingly, retraction bulbs showed an intense LC3 staining in sections 0.5–1 mm around the injury epicenter (Figure 4D). In the gray matter, LC3 signal of the neuropil was reduced, particularly in the dorsal horns. Neither neurons nor oligodendrocytes or astrocytes exhibited important changes respect to undamaged spinal cords.

**Figure 5:**
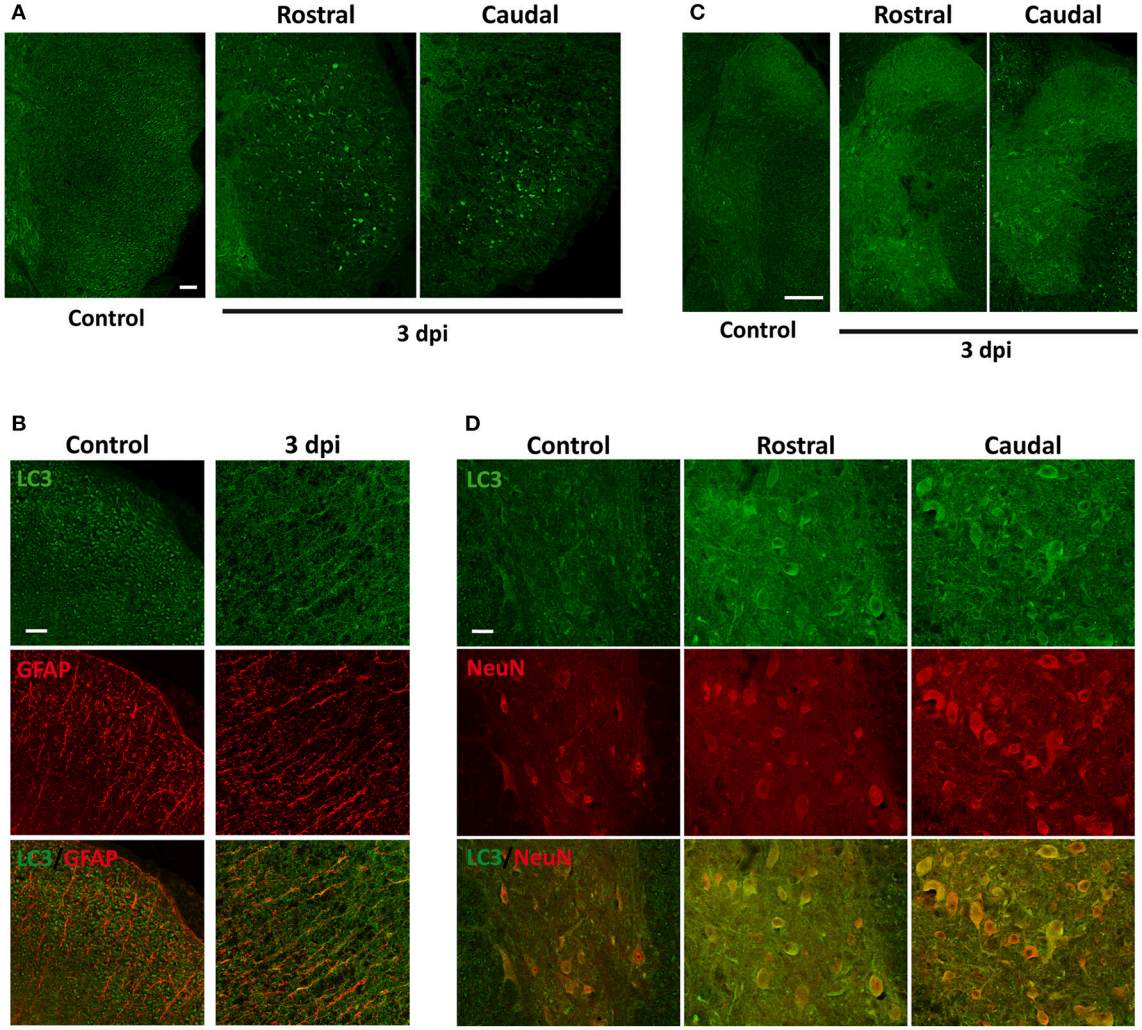
LC3 expression increases 3 days after injury (3 dpi), especially in astrocytes. **(A)** Representative confocal images coronal sections of non-injured (left) and 3 dpi (middle and right) wild-type mouse spinal cords labeled with anti-LC3 antibody (*n* = 3 per condition). 3 dpi images correspond to sections rostral (middle) and caudal (right) to the injury epicenter. **(B)** Detail of the lateral white matter of non-injured (left) and 3 dpi spinal cord sections (right) labeled with anti-LC3 (upper panels) and anti-GFAP (astrocyte marker) (middle) and the merged co-expression (bottom). **(C)** Detail of the gray matter of non-injured (left) and 3 dpi sections rostral (middle) and caudal (right) from the epicenter showing anti-LC3 labeling. **(D)** High magnification confocal images of non-injured (left) and 3 dpi rostral (middle) and caudal (right) sections labeled with anti-LC3 (upper panels), anti-NeuN (middle panels) or both (bottom panels). Images of injured spinal cords were obtained from sections 0.5–1 mm away from the epicenter in rostral or caudal directions. Scale bars: 100 μm in **(A)**, 50 μm in **(B,D)**, and 150 μm in **(C)**.

At 3 dpi, spinal cords showed a slight recovery of the LC3 signal, particularly in rostral sections up to 3 mm away from the injury (Figure 3). In the white matter, the number of tracts continued decreasing and so the associated LC3 signal. This trend was particularly evident in caudal sections up to 3 mm away from the epicenter, whereas rostral sections preserved stained tracts and retraction bulbs in the dorsal and lateral *funiculi* (Figure 5A). On the contrary, LC3 staining increased in astrocytes within a 1 mm range away from the injury epicenter (Figure 5B). In the gray matter, global LC3 labeling increased (Figure 5C) and some neurons in the ventral horn and the region surrounding the ependyma exhibited strong LC3 staining in sections 0.5 to 1 mm around the injury (Figure 5D).

**Figure 6:**
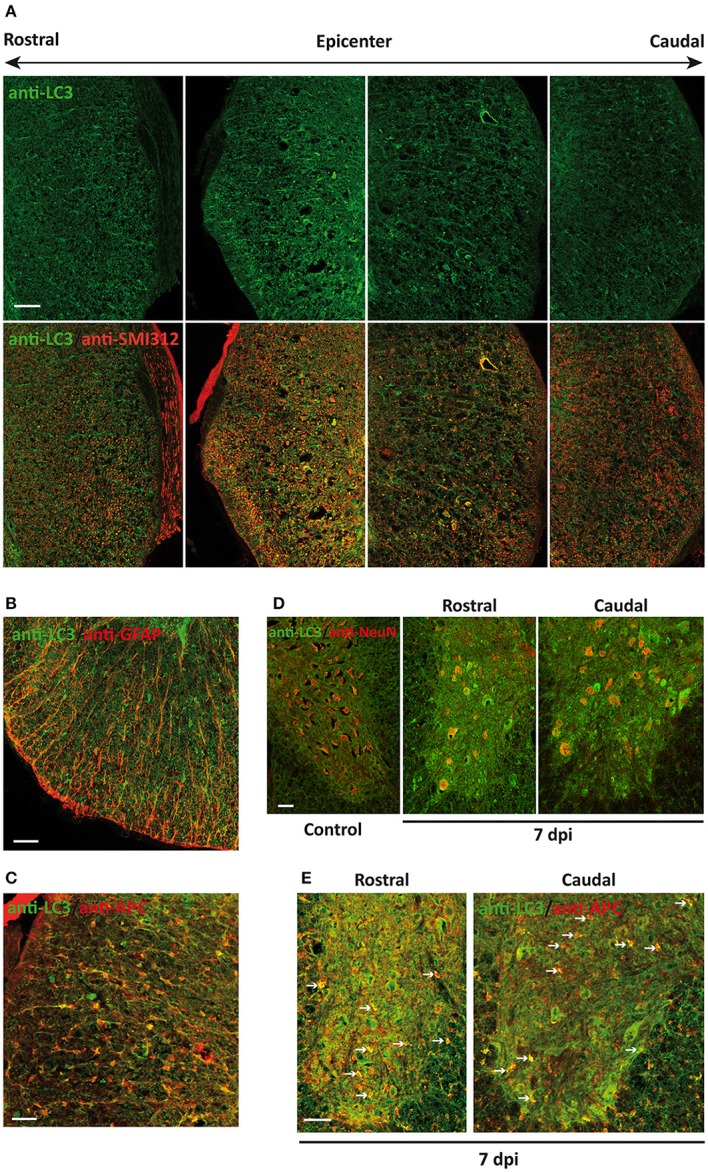
Neural cells of the spinal cord reach maximum LC3 expression 7 days after injury (7 dpi). **(A)** Representative confocal images of coronal sections of 7 dpi spinal cord from wild-type mice labeled with anti-LC3 (upper panel) and its co-expression with anti-SMI-312 (axonal marker) (bottom panel) (*n* = 3). Images were obtained at different distances from injury epicenter, from 3 mm rostral (left) to 3 mm caudal (right). **(B,C)** Representative confocal images of sections from 7 dpi white matter co-stained with anti-LC3 and cellular markers anti-GFAP (astrocytes, **B**) and anti-APC (oligodendrocytes, **C**). **(D)** Representative confocal images of non-injured (left) and 7 dpi sections co-labeled with anti-LC3 and neuronal marker anti-NeuN antibodies. **(E)** Representative confocal images of non-injured (left) and 7 dpi gray matter sections co-labeled with anti-LC3 and oligodendroglial marker anti-APC antibodies. In both **(D,E)**, images from 7 dpi spinal cords were acquired from sections 0.5–1 mm rostral (middle) and caudal (right) to the injury epicenter (*n* = 3). Farthest sections are separated about 3 mm and nearest ones about 0.5–1 mm from injury epicenter. Scale bars: 150 μm in **(A)** and 50 μm to **(B–E)** *n* = 3.

At 7 dpi, LC3 signal became more intense, resembling that of control sections (Figure 3) but with a very different pattern. In the white matter, overall LC3 signal was lower than in controls. High magnification analyses revealed that LC3 reduction was due to the loss of axonal tracts, particularly in sections caudal to the injury site (Figure 6A). However, LC3 staining increased strongly in astrocytes (Figure 6B) and oligodendrocytes (Figure 6C) through all sampled tissue. In the gray matter, the general intensity of LC3 staining became higher than in controls (Figure 6D), with many strongly stained neurons in all sampled tissue (Figure 6E). Some oligodendrocytes from the gray substance were also strongly stained in the proximities (0.5–1 mm) to the injury epicenter (arrows in Figure 6E). This staining pattern was preserved at 14 dpi, although signal intensity in the sections caudal to the injury site was lower than in corresponding 7 dpi sections.

**Figure 7:**
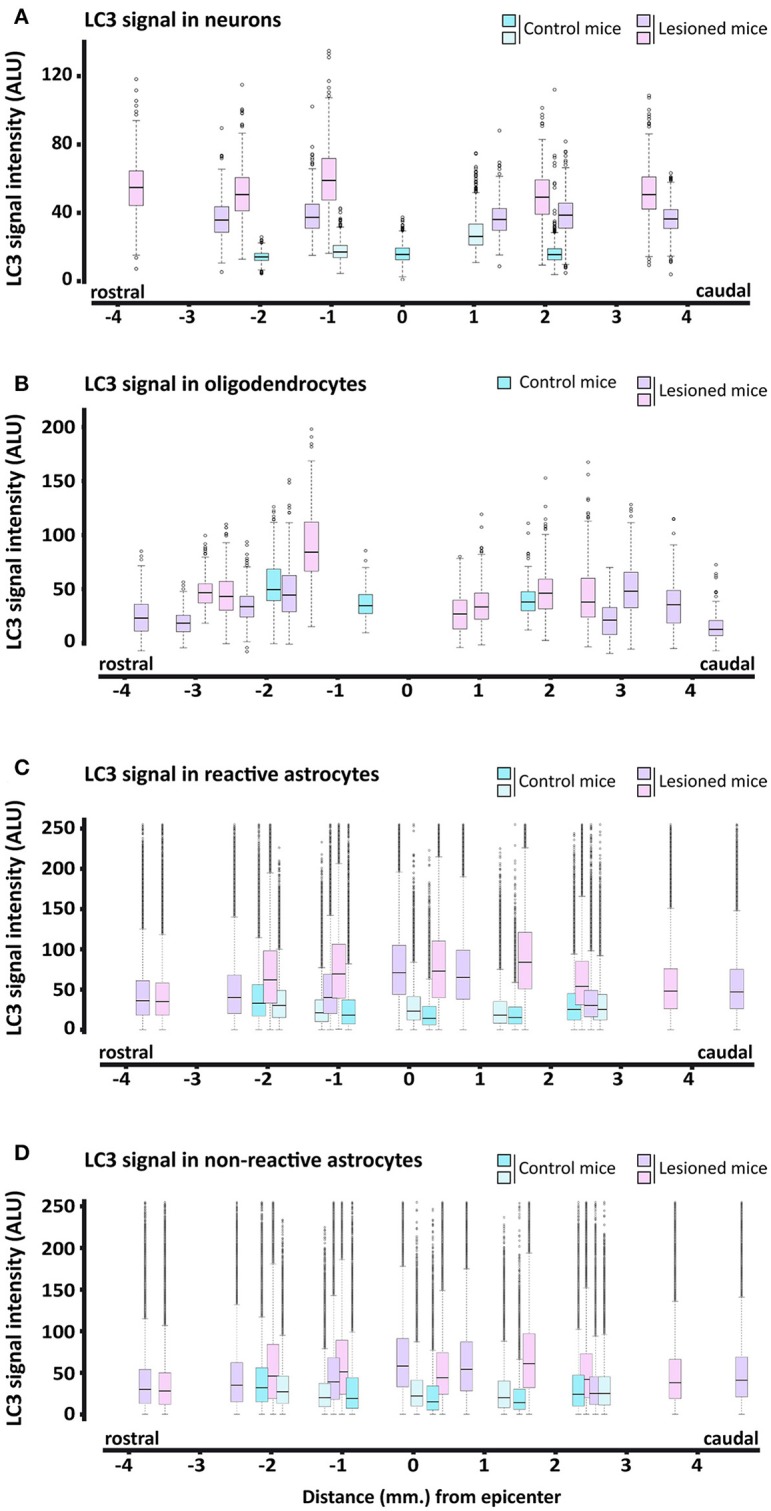
Quantification of LC3 expression in neural cells of 7 dpi spinal cords. Intensity of anti-LC3 signal was measured from immunofluorescence confocal images of coronal sections of control (blue) and 7 dpi (pink/purple) spinal cords from wild-type mice. Quantification methods for each cell type are described in the methods sections. Signal intensity is represented through box and whisker plots at different spatial positions relative to the injury epicenter in neurons (anti-NeuN) **(A)**, oligodendrocytes (anti-APC), **(B)** and reactive **(C)** and non-reactive astrocytes (anti-GFAP) **(D)**. Each box and whisker represents the LC3 signal intensities in all cells measured from a specific section (with a specific position relative to the injury epicenter) of a control or 7 dpi spinal cord. For each individual (*n* = 2), we analyzed 3–4 sections at increasing distances rostral and caudal to the injury epicenter. Approximately 800 neurons and 80–70 oligodendrocytes were analyzed in each section. To estimate LC3 expression in reactive and non-reactive astrocytes, we measured LC3 signal in individual pixels with GFAP signal intensity between 50 and 100 and above 100, respectively.

Quantitative analysis of individual cells from complete sections of undamaged and 7 dpi spinal cords revealed that LC3 staining in neurons was higher in all injured sections than in undamaged ones (Figure 7A). LC3 signal in oligodendrocytes was also higher at 7 dpi, particularly in those sections in the first 2 mm rostral to the injury epicenter (Figure 7B). In astrocytes, LC3-signal at 7 dpi was higher only in GFAP positive pixels (see Figures 7C,D, respectively) from sections located in the 2.5 mm surrounding the epicenter. LC3 signal was stronger and more widely distributed in pixels with high GFAP signal (associated with reactive astrocytes) than in low GFAP ones.

**Figure 8:**
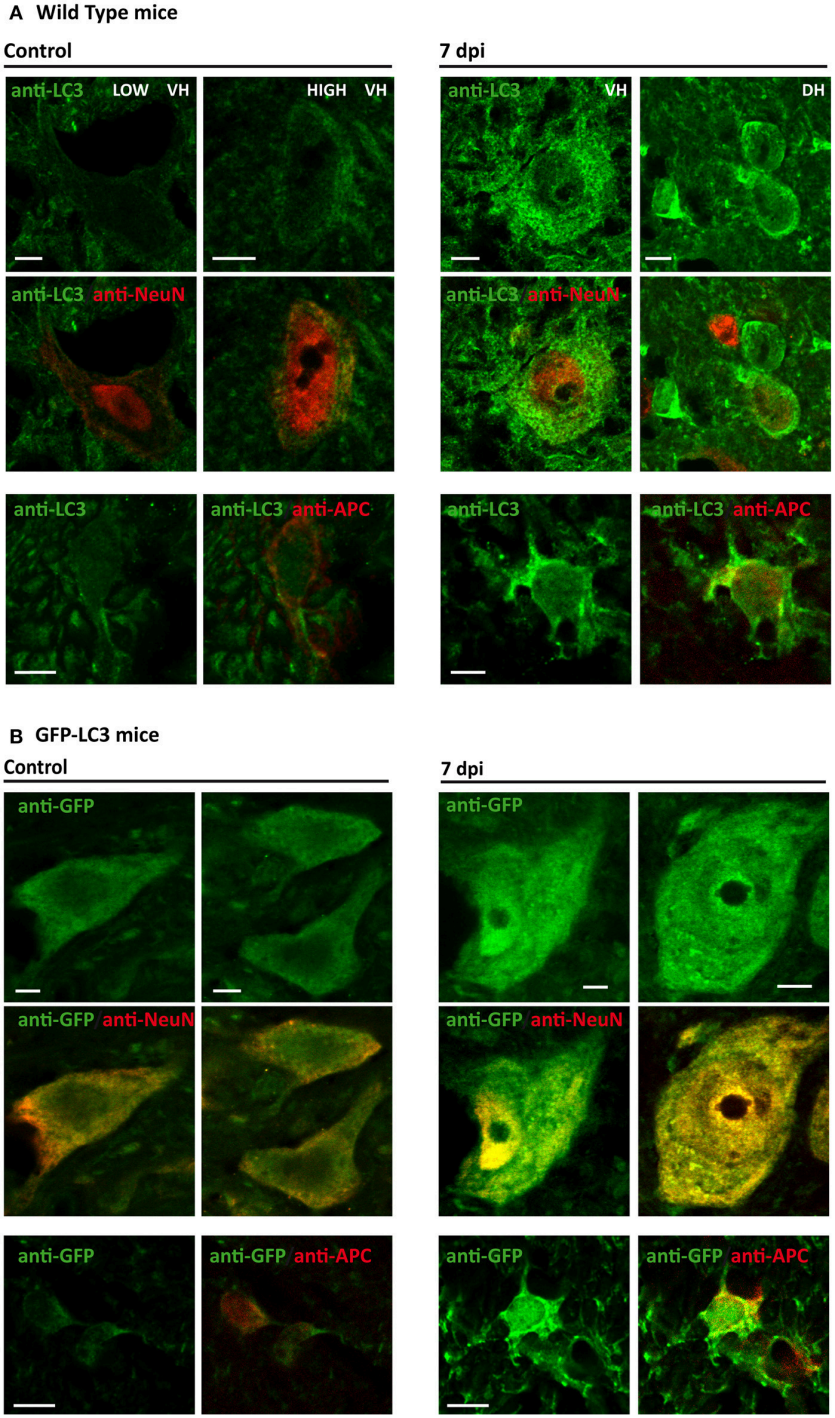
The number of autophagosomes increases in 7 dpi spinal cords. Comparison of LC3 expression in control (left panels) and 7 dpi (right panels) spinal cords from wild-type **(A)** and GFP-LC3 transgenic mice **(B)** (*n* = 3). In both cases, upper and middle panels shows LC3 expression in neurons (marked with anti-NeuN marker), whereas the bottom panels show LC3 expression in oligodendrocytes (anti-APC). VH indicates ventral horn, whereas DH indicates dorsal horn. In **(B)**, LC3 was indirectly stained using an antibody against GFP. Single plane (0.5 μm thick) high magnification images of coronal sections of the spinal cord were acquired employing a confocal microscope equipped with a 63 × objective with a 3.5X digital zoom. Scale bar: 5 μm.

According to previous studies, LC3 staining pattern can be used to differentiate LC3-I (diffuse pattern) from autophagosome-associated LC3-II (dot pattern) at appropriate conditions (Mizushima et al., 2010; Klionsky et al., 2016). Considering that immunoblotting revealed an important increase of LC3-II protein at 7dpi, we used high magnification confocal images to analyze the pattern of LC3 staining at sub-cellular level in control and 7 dpi sections. The so-obtained images revealed that most neurons from both ventral and dorsal horns of non-injured spinal cords presented a low and predominantly diffuse staining in the cytoplasm (Figure 8A, left upper panel). A few neurons from Redex's VII, VIII, and IX laminae showed a stronger signal associated with LC3-possitive puncta that would correspond to autophagic structures. Oligodendrocytes from undamaged spinal cord sections also exhibited a similar low and disperse cytoplasmatic LC3 signal, although a few LC3 dots were also observed in processes near the soma of some cells (Figure 8A, left lower panel). At 7 dpi, the staining pattern changed in both cell types. Overall, neurons presented an increase of LC3 signal in the cytoplasm, in many cases showing a dot pattern (Figure 8A, right upper panel). Likewise, all examined 7 dpi oligodendrocytes showed a dot LC3 staining that spread from the cellular processes to their soma (Figure 8A, right lower panel). Analyses of spinal cord samples from GFP-LC3 transgenic mice, presenting a lower signal background, confirmed that the increase in LC3 staining at 7 dpi was associated with the presence of more LC3 dots in the analyzed neurons (Figure 8B, upper panel). In the case of oligodendrocytes, un-injured spinal cords presented low staining with few puncta restricted to their processes, whereas in 7 dpi spinal cord sections, intensity of LC3 staining increased strongly in the cytoplasm with LC3 dot pattern spanning from the proximal processes to the oligodendrocyte soma (Figure 8B, lower panel). Therefore, these results suggest that autophagosome abundance rises in neurons and oligodendrocytes at 7 dpi.

### Is the increase of autophagosome abundance following SCI associated with an initiation of autophagy?

**Figure 9:**
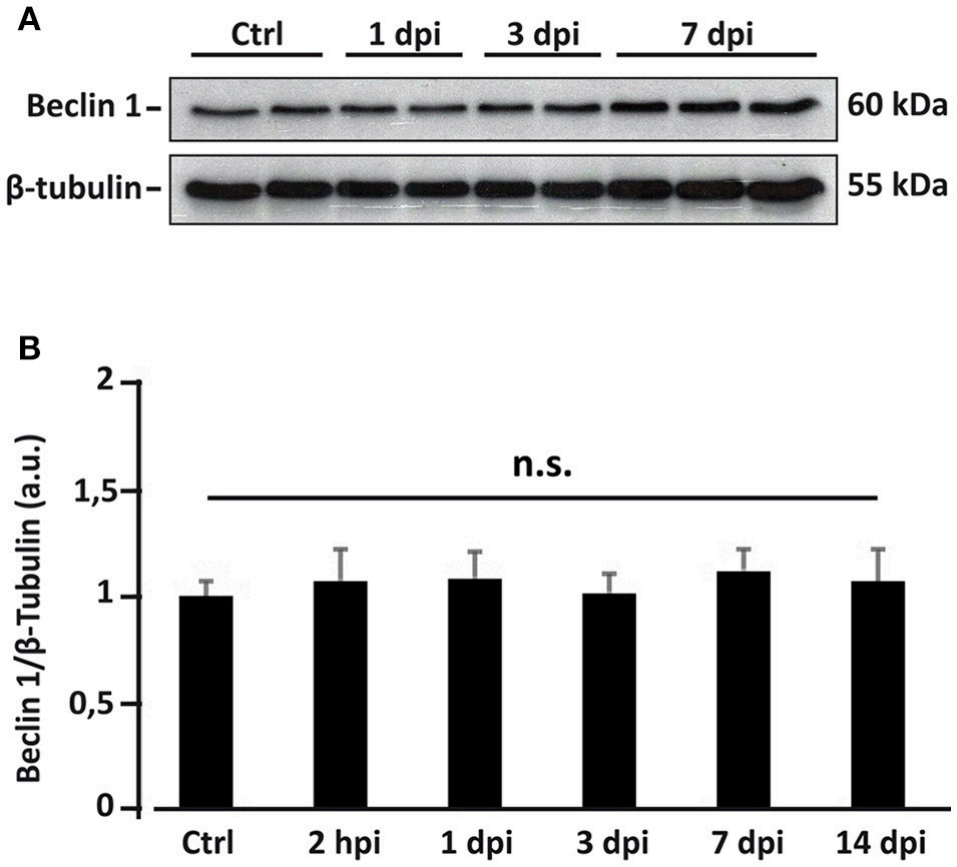
Overall Beclin 1 expression does not change after spinal cord injury. **(A)** Representative western blot of spinal cord lysates from control and injured spinal cords sampled at different post-injury times (each lane represents an individual). **(B)** Densitometric analysis of Beclin 1 bands from spinal cord homogenates sampled at 2 hpi (*n* = 5), 1 (*n* = 5), 3 (*n* = 5), 7 (*n* = 5), and 14 dpi (*n* = 3), and non-injured controls (*n* = 6). Data were normalized to β-tubulin and then relativized to the mean of the control values. Bars represent mean ± S.E.M. n.s indicates non-significant differences (*p* > 0.05) according to one-way ANOVA. The original unprocessed scans employed in this figure are available in the Figure S7.

Rise in autophagosome abundance (inferred from the LC3 data) following SCI may result from an enhanced initiation of the autophagic flux (Rubinsztein et al., 2009), that would be related to an increased expression of Beclin 1. To test this hypothesis, we first analyzed the expression levels of Beclin 1 by inmunoblotting of protein samples from control, 2 hpi, 1, 3, 7, and 14 dpi spinal cords (Figure 9A). No significant changes in the levels of Beclin 1 were observed at any time after injury (one-way ANOVA, *F*_(5, 25)_ = 0.8811; *p* = 0.5081) (Figure 9B), suggesting that autophagic initiation does not change during the first days after injury, and therefore is not the main factor for the observed augment in autophagic structures abundance.

**Figure 10:**
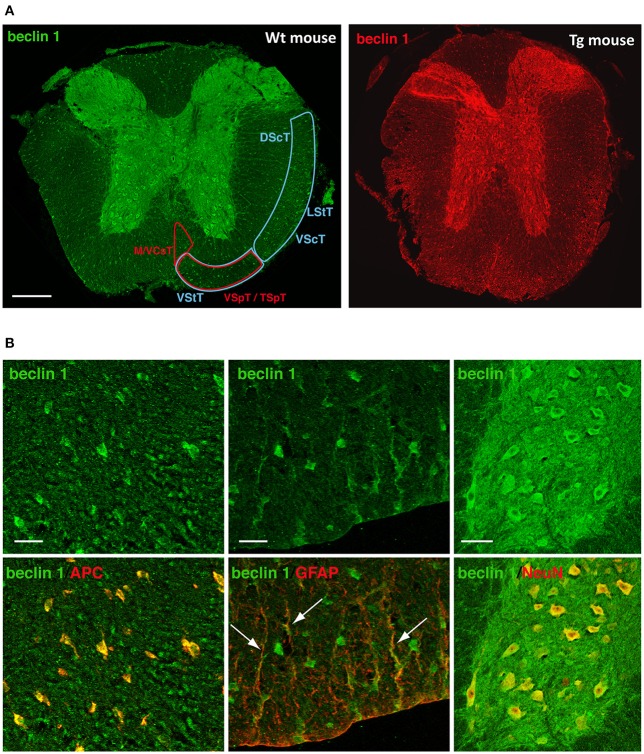
Beclin 1 expression in neural cells of the undamaged spinal cord. **(A)** Representative images of Beclin 1 labeling in coronal sections of control spinal cords from wild-type (left) and GFP-LC3 transgenic mice (right) (*n* = 3). Left panel shows Beclin 1 labeling in both ascending (blue areas) and descending (red areas) tracts. **(B)** Representative high magnification images of Beclin 1 labeling (upper panels) in coronal sections of control spinal cords from wild-type mice and co-labeling (lower panel) with cellular markers anti-APC (oligodendrocytes, left), anti-GFAP (astrocytes, middle) and anti-NeuN (neurons, right). **(A)** abbreviations correspond to: DScT, dorsal spinocerebelar tract; LStT, lateral spinothalamic tract; VScT, ventral spinocerebelar tract; VStT, ventral spinothalamic tract; M/VCsT, medial and ventral corticospinal tract; VSpT, vestibulospinal tract; TSpT, tectospinal tract. Scale bar correspond to 500 μm in **(A)**; in **(B)**, bars correspond to 50 μm in oligodendrocyte and astrocyte panels, and to 25 μm in neuron panel. White arrows point to GFAP and Beclin-1 co-staining, that is, Beclin-1 positive astrocytes.

Immunofluorescence imaging provided complementary spatiotemporal and cell information. Undamaged spinal cords exhibited high Beclin 1 signal in various structures and neural cell types. In the white matter, the main ascending tracts –dorsal, lateral and ventral spinocerebellar tracts- and descending tracts –medial and anterior corticospinal and vestibulo/tectospinal tracts—(Figure 10A), as well as oligodendrocytes showed Beclin 1 immunoreactivity (Figure 10B, left image). Conversely, there were only a few Beclin 1 positive astrocytes, restricted to those in contact with the meninges (Figure 10B, medium image). In the gray matter, we observed a strong Beclin 1 staining in the neuronal somas, as well as in the neuropil (Figure 10B, right image).

**Figure 11:**
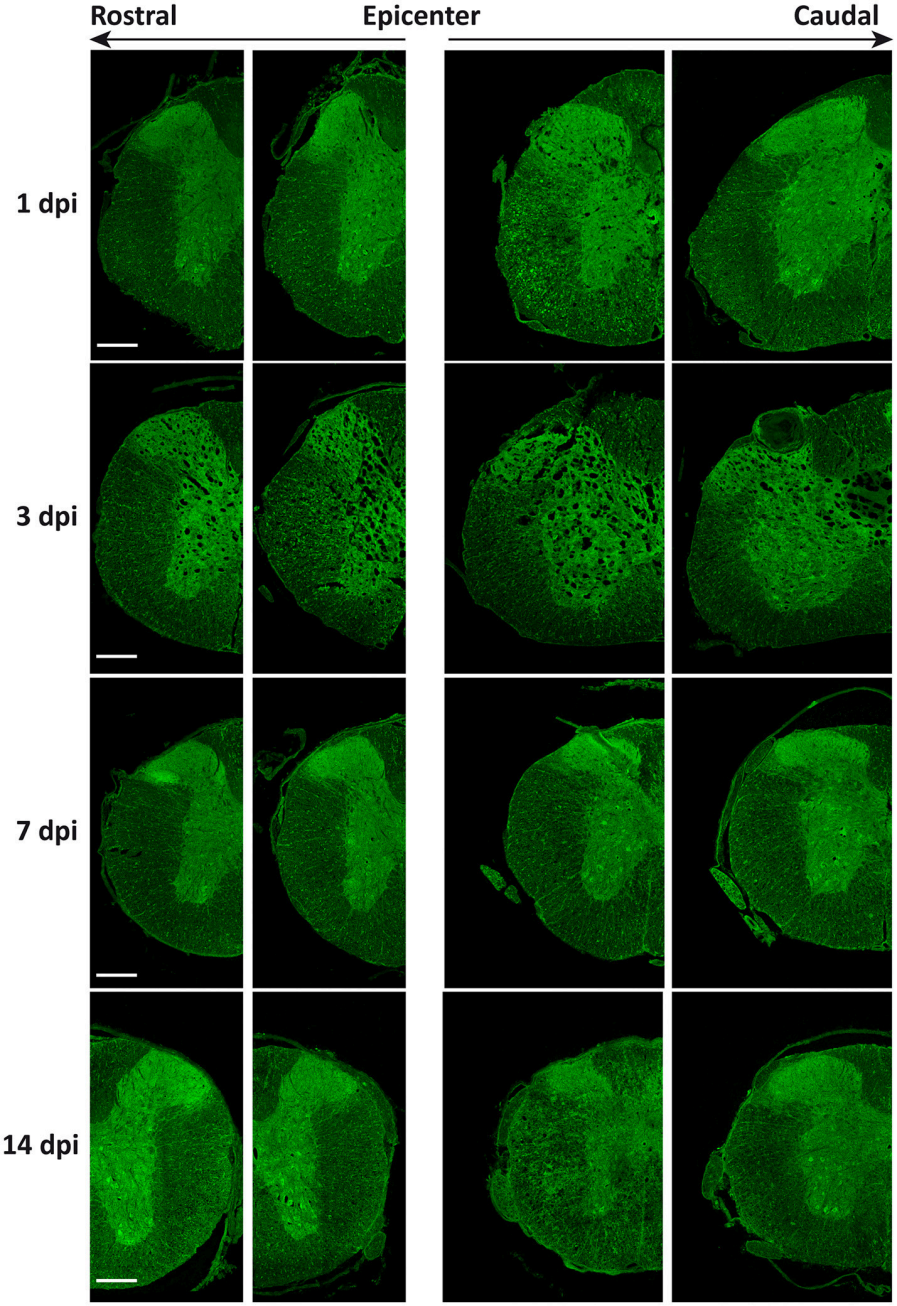
General distribution of Beclin 1 expression does not change after spinal cord injury. Representative images of coronal sections from wild-type mouse spinal cords labeled with anti-Beclin 1 antibody at the indicated time points after SCI (from top to bottom) and the increasing distances from the injury epicenter (from left to right) (*n* = 3 individuals per time point). Farthest sections are approximately 3 mm away from the injury epicenter whereas nearest sections are only 0.5 to 1 mm away. Scale bar corresponds to 500 μm.

**Figure 12:**
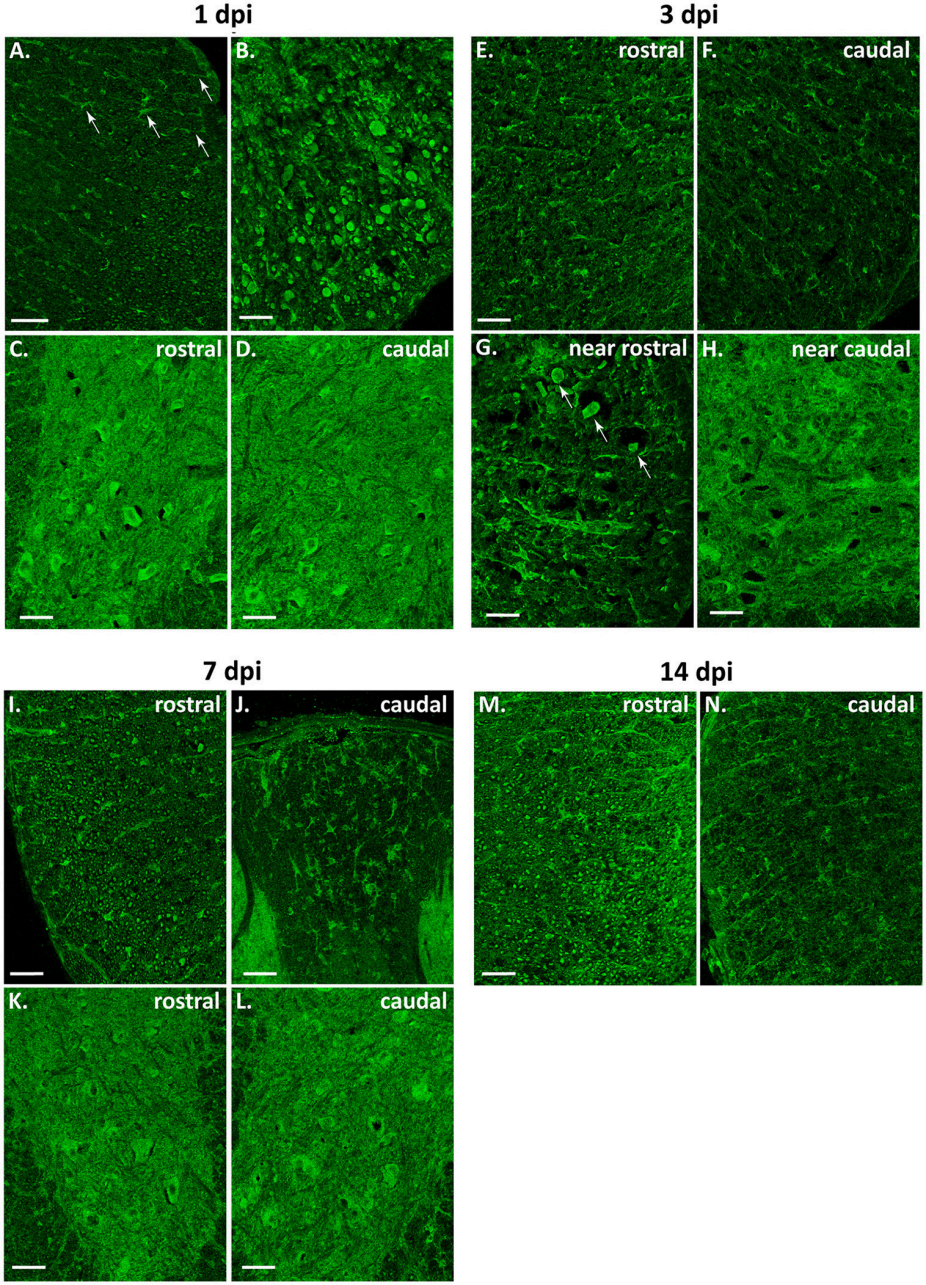
Beclin 1 expression increases in astrocytes but not in neurons or oligodendrocytes after spinal cord injury. Representative magnified images of coronal sections of 1–14 dpi spinal cords from wild-type mice labeled with anti-Beclin 1 antibody (*n* = 3 individuals per time point). **(A–D)** 1 dpi Beclin 1 expression in astrocytes (**A**, white arrows) and retraction bulbs **(B)** from the white matter (WM), and in neurons from regions of the gray matter (GM) rostral **(C)** and caudal **(D)** to the injury epicenter. **(E–H)** 3 dpi Beclin 1 expression in WM **(E,F)**, in retraction bulbs from regions of the WM rostral to the injury site (**G**, white arrows), and in neurons from caudal regions **(H)**. **(I–L)** 7 dpi Beclin 1 expression in tracts from sections rostral to the injury site **(I)**, in astrocytes from caudal sections **(J)** and in neurons from rostral **(K)** and caudal **(L)** sections. **(M–N)** 14 dpi Beclin 1 expression in astrocytes from sections rostral to the injury **(M)** and in axonal tracts from caudal sections **(N)**. Rostral and caudal images correspond to sections 0.5 to 1 mm away from the injury site. Scale bars: 50 μm in **(A)**; 100 μm in the rest of images.

As shown in Figure 11, global intensity of Beclin 1 signal did not greatly differ among spinal cord sections from the different time conditions, in agreement with immunoblotting results (Figure 9A). However, a more detailed observation revealed marked differences in the Beclin 1 staining levels among neural cell types, dpis, and locations. At 1dpi, overall Beclin 1 intensity remained similar to controls in the white matter (Figure 12, 1 dpi panel), although Beclin 1-positive astrocytes and retraction bulbs appeared in sections 0.5–1 mm away from the epicenter (Figures 12A,B, respectively). In the gray matter, Beclin 1 staining remained intense, but many neuronal somas showed lower Beclin 1 staining than in control sections. In fact, a few neurons from Redex's laminae VII and IX in rostral sections and in those caudal sections located 0.5–1 mm away from the injury epicenter showed strong Beclin 1 staining (Figures 12C,D, respectively).

In 3 dpi spinal cords, loss of tracts reduced Beclin 1 signal in the white matter, particularly in sections caudal to the injury site (Figures 12E,F). Beclin 1 staining was clearly visible among swelling axons from sections 0.5–1 mm rostral to the injury (Figure 12G). Oligodendrocytes showed lower staining than in control and 1dpi sections, whereas astrocytes kept on increasing their Beclin 1 signal. In the gray matter, there were no changes in neuropil staining, whereas Beclin 1 intensity decreased in neuronal somas (Figure 12H).

At 7 dpi (Figure 12, 7 dpi panel), there was a slight recovery in the staining of the preserved tracts, especially in regions rostral to the injury (Figure 12I). As observed at 3 dpi, oligodendrocytes showed a low Beclin 1 signal whereas astrocytes showed high staining in all analyzed sections (Figure 12J). In the gray matter, some neurons exhibited high Beclin 1 staining, particularly in caudal sections (Figures 12K,L).

Finally, at 14 dpi (Figure 12, 14 dpi panel), Beclin 1 signal increased in tracts from regions rostral to the injury, almost resembling control sections. This pattern was not observed in sections caudal to the injury, due to the general loss of axonal tracts (Figures 12M,N). Beclin 1 signal also increased in white matter astrocytes. Oligodendrocytes maintained a low Beclin 1 signal in all sampled sections. In the gray matter, neuronal somas showed a Beclin 1 staining similar to that observed at 7 dpi.

**Figure 13:**
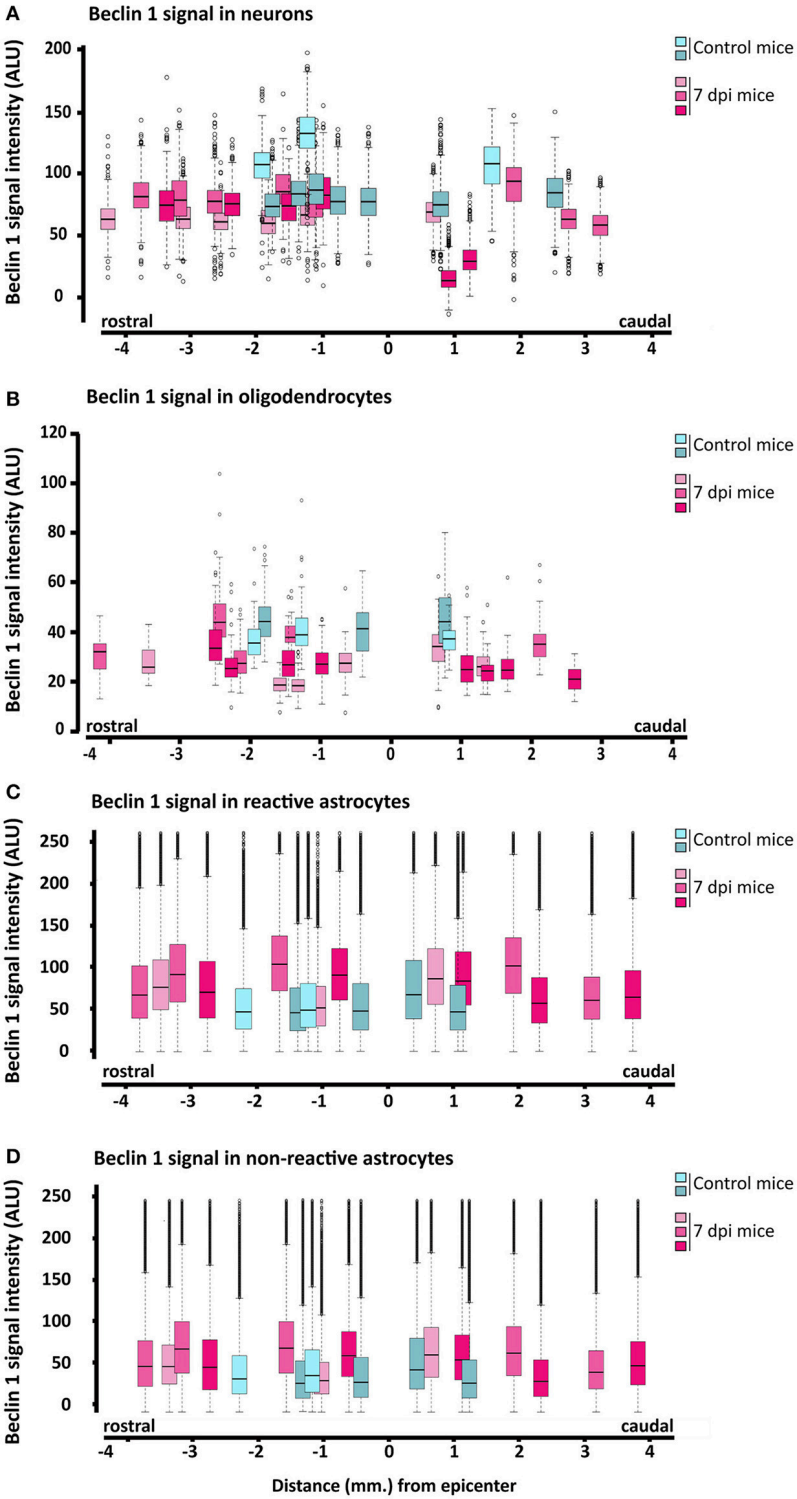
Quantification of Beclin 1 expression in neural cells from 7 dpi spinal cords. Intensity of Beclin 1 immunofluorescence was measured in confocal images of control (blues) and 7 dpi (pinks) spinal cord coronal sections from wild-type mice. Quantification procedures for each cell type are described in the methods section. Signal intensity for each cell type (**A**, neurons; **B**, oligodendrocytes; **C**, reactive astrocytes, and **D**, for resting astrocytes) at increasing distances to the injury epicenter is represented through individual box and whisker plots, each one representing the Beclin-1 signal intensities in all cells measured from a specific section (with a specific position relative to the injury epicenter) of a control or 7 dpi spinal cord. In each spinal cord (*n* = 2, control mice; *n* = 3, injured mice), we analyzed 3–4 sections in both rostral and caudal directions from the injury epicenter. Signal intensity of Beclin 1 was measured in approximately 800 neurons and 80–70 oligodendrocytes per spinal cord section. To estimate Beclin-1 expression in reactive and non-reactive astrocytes, we measured Beclin-1 signal in individual pixels with GFAP signal intensity between 50 and 100 and above 100, respectively.

To further explore the changes in Beclin 1 expression, we quantified the intensity of Beclin 1 staining in sections of 7 dpi spinal cords, when autophagosome abundance is at its highest according to our previous results, and referred to non-injured ones. In these analyses, we followed the same procedures that we employed to quantify LC3 signal. According to the so-obtained data (Figures 13A,B), the intensity of Beclin 1 staining did not exhibit any clear spatial variation in neurons and oligodendrocytes from 7 dpi sections, resembling non-injured spinal cords. Conversely, astrocytes analyses revealed that the intensity of Beclin 1 labeling in GFAP positive pixels was higher in 7 dpi spinal cords than in control ones (Figures 13C,D), particularly in those pixels with high GFAP intensity (i.e., corresponding to reactive astrocytes). The increase in Beclin 1 intensity among GFAP positive pixels remained similar across all 7dpi sections, without differences in the tissue surrounding the injury site.

**Figure 14:**
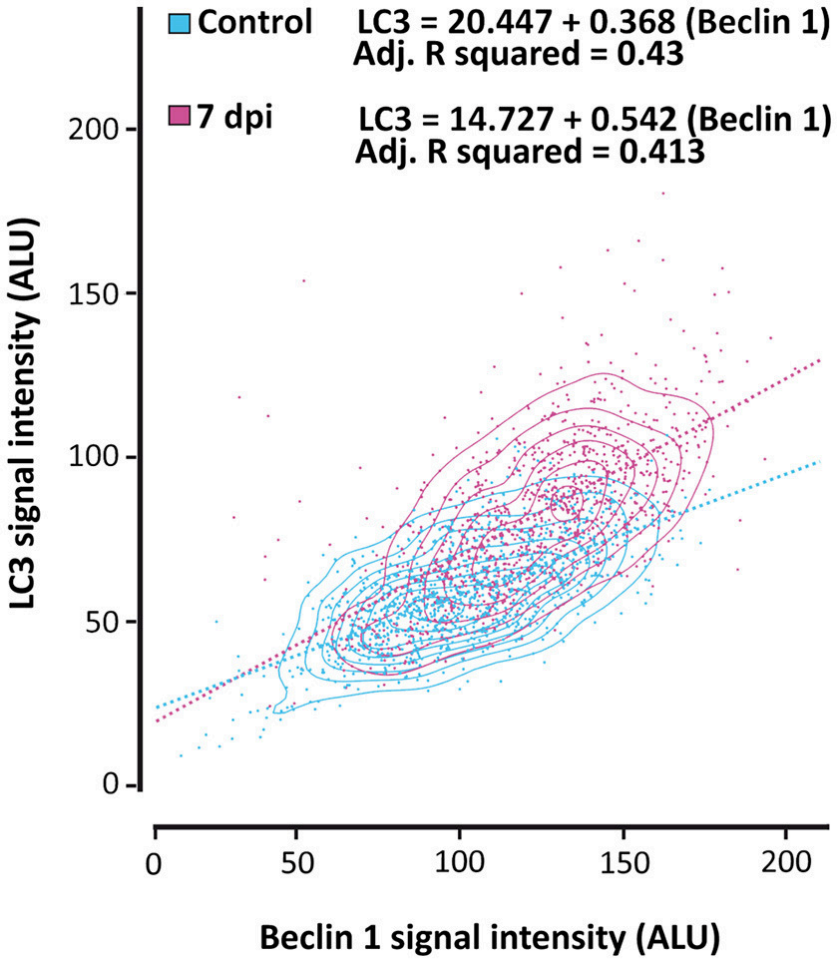
The relationship between LC3 and Beclin 1 expression in neurons changes after SCI. LC3 and Beclin 1 expression in neurons (stained with anti-NeuN) was estimated in confocal images of spinal cord sections from control and 7 dpi GFP-LC3 mice labeled with anti-GFP (for LC3 detection) and anti-Beclin 1. The graphic shows the relationship between expression levels of both proteins in neurons from control (blue) and 7 dpi (pink) spinal cords. Approximately 800 neurons were analyzed for each mouse (*n* = 2 per time point).

To specifically analyze the relationship between the expression of LC3 and Beclin 1 in neurons and how it changes after SCI, we quantified the intensity of LC3 and Beclin 1 signals in 70–80 NeuN-stained neuronal somas per section (5–9 sections per individual; 350–700 neurons/individual). We carried out these analyses in non-injured and 7 dpi spinal cords from GFP-LC3 mice, which showed lower background signal than wild type animals. As illustrated in Figure 14, (for analysis see Figure S4) the obtained data demonstrated that LC3 and Beclin 1 staining levels are related, both before and after injury, although Beclin 1 intensity only explained a roughly 40% of the variation in LC3 intensity (Adj R-squared in control samples = 0,43; in injured sample = 0,413), leaving nearly a 60% subjected to other factors. Interestingly, the slope of the regression lines from control and 7dpi samples differed significantly (*p* < 0.001), being markedly higher in 7 dpi (slope = 0.542) than in controls (slope = 0.368), that is, similar levels of Beclin 1 would result in higher LC3 staining in 7 dpi samples than in control ones. These results suggest that a given increase of autophagic initiation results in a much higher autophagosome abundance in 7dpi spinal cords than in control ones.

All-together, results obtained from our immunoblotting and immunofluorescence analyses revealed that Beclin 1 expression only raises in astrocytes, in parallel to the observed increase in LC3 expression, suggesting that the increase in the abundance of autophagic structures among astrocytes is likely due to an enhanced initiation. On the contrary, Beclin 1 expression in neuronal somas and oligodendrocytes remained almost unchanged after SCI, suggesting that the increase of LC3 levels observed in these cells is not caused by autophagy initiation.

**Figure 15:**
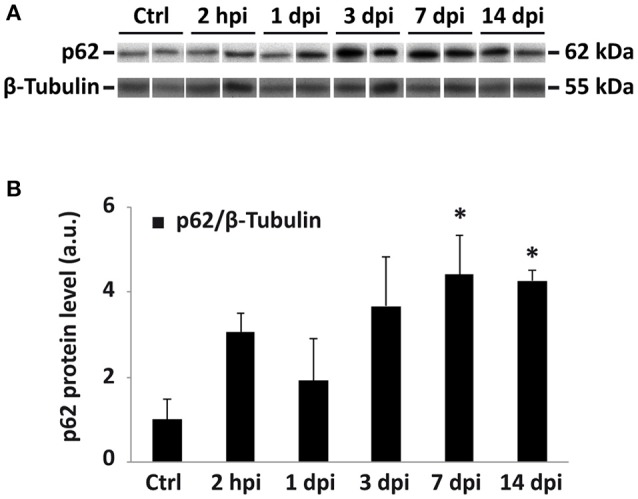
p62 protein accumulation after spinal cord injury suggest a possible blockage of the autophagic flux. **(A)** Immunoblot of p62 in lysates from control and different dpi spinal cords. Each lane corresponds to an individual animal. Only a representative subset of the analyses samples is represented (for the complete set see Figure S5). **(B)** Densitometric analysis of p62 bands at different times after injury (*n* = 6 for controls; *n* = 5 for 2 hpi; *n* = 4 for 1 dpi; *n* = 3 for 3 and 7 dpi; and *n* = 4 for 14 dpi). Data were normalized to tubulin and relativized to the mean of control values. Bars represent mean ± S.E.M; * indicates *p* < 0.05. Probabilities estimated from one-way ANOVA followed by Dunnett's *post-hoc* test.

Alternatively, autophagic structures accumulation among these cells may result from an impairment in autophagosome degradation, as already proposed by Liu et al. (Liu et al., 2015) in a rat model of SCI. To test this hypothesis, we evaluated the expression of SQSTM-1/p62 protein (hereafter referred as p62 protein), which recruits ubiquitinated proteins, organelles and noxious protein aggregates and delivers them to autophagosomes for degradation (Pankiv et al., 2007). Moreover, p62 is itself a substrate of autophagy, that becomes efficiently degraded after binding to LC3 (Bjørkøy et al., 2005). As a consequence, p62 accumulates in the cell when autophagosome degradation is blocked (Ichimura et al., 2008). We evaluated p62 expression by immunoblotting of protein samples from non-injured and 2 hpi, 1, 3, 7, and 14 dpi spinal cords (Figure 15A; Figure S5). Resulting data revealed that levels of p62 gradually increase after injury, peaking at 7 dpi (one-way ANOVA, *F*_(5, 20)_ = 3,22; *p* < 0,05; Dunnett's Multiple Comparison test, *p* < 0,05), and remain high at 14 days after the trauma (Figure 15B). We are aware that immunofluorescence studies are needed to confirm this finding, and to identify which cell types are responsible for this p62 accumulation. However, present p62 results agree with a possible blockage of the autophagic flux that would account for the increase of LC3 staining in neurons and oligodendrocytes.

## Discussion

Recently described alterations of the autophagic process during the course of SCI (Kanno et al., 2009a, 2011; Tanabe et al., 2011; Zhang et al., 2013a, 2014; Hou et al., 2014; Liu et al., 2015; Ribas et al., 2015) may constitute interesting targets for therapy development. However, despite a general agreement on autophagy alteration, consensus lacks in almost every detail, from the nature and timing of the changes to the affected cells or the involved mechanisms. We have tried to clarify these details, characterizing autophagy in a model of contusive SCI (Metz et al., 2000; Young, 2002), a particularly relevant model considering the high prevalence of contusions among SCI patients.

### Autophagy in the undamaged spinal cord

Despite the existence of several analyses of autophagy in experimental spinal cord injuries, little is known on the basal state of autophagy in the undamaged spinal cord. Our immunoblotting analyses revealed detectable levels of LC3-II levels, evidencing the presence of autophagic structures and confirming that autophagy is constitutively active in the undamaged spinal cord.

Immunofluorescence analyses provided additional details on the cell and tissue distribution of autophagy and revealed clear differences among the main neural cells of the spinal cord. In neurons, LC3 staining is strong in most tracts but is much more variable in the somas. According to Sandra Maday and coworkers (Maday and Holzbaur, 2012, 2014; Maday et al., 2012, 2014; Maday, 2016), high LC3 staining in the tracts reflects the enhanced initiation of autophagosome formation in the distal segment of the axons (also confirmed by Beclin-1 staining) and their retrograde transport toward the soma. The comparatively low LC3 signal observed in the soma of many neurons (also described in ref. Lee, 2012) would be explained by the efficient degradation of the transported autophagosomes, as demonstrated by experiments inhibiting lysosome activity (Boland et al., 2008). However, strong LC3 staining and the presence of LC3-positive puncta in a number of neuronal somas indicate that some neurons accumulate autophagic structures in their cell bodies. Although this observation contrasts with results from early *in vivo* and *in vitro* studies, which hardly detected autophagosomes among healthy neurons (Nixon et al., 2000; Mizushima et al., 2004), it resembles observations from more recent reports, including specific studies in mouse spinal cord that identified LC3 vesicles in almost a 30% of ventral horn motoneurons (Alirezaei et al., 2010; Castillo et al., 2013). This variability may be explained, at least in part, by differences in the extent of autophagic initiation at the soma, a process previously described by Maday (Maday and Holzbaur, 2016).

Basal autophagy among spinal cord oligodendrocytes somehow resembles that of neurons. Oligodendrocytes present a highly heterogeneous LC3 staining both in wild type and GFP-LC3 mice, with many cells almost lacking any detectable signal whereas others present autophagic structures in the processes near the cell body, a pattern previously described by Schwarz and co-workers (see ref. Schwarz et al., 2012, Figures 6B, 7A) in primary cultures of oligodendrocytes from rat brain. Variability in the abundance of autophagic structures seems to be the rule in the whole CNS according to previous descriptions in rat spinal cords (see ref. Liu et al., 2015, Figures 4E,F) and human brains (Satoh et al., 2014) but contrasts with the sustained initiation of the autophagic process in these glial cells inferred from the high Beclin-1 staining described here as well as in human and rodent adult brain (Liang et al., 1998; Yue et al., 2002; Carloni et al., 2008; Rami et al., 2008). Therefore, lack of LC3 in many oligodendrocytes does not imply the absence of autophagic activity. On the contrary, constitutive autophagy seems to be required for both neuron and oligodendrocyte survival and function, as evidenced by studies with knockout rodents in which lack of autophagic proteins disrupts neural function, leading to neuron and oligodendrocytes degeneration (Hara et al., 2006; Komatsu et al., 2007; Smith et al., 2013; Bercury et al., 2014).

In astrocytes, autophagic structures seem to be scarce. In fact, astrocytes lack detectable LC3 staining in all regions of the undamaged spinal cord, except in those cells at the boundary with the *pia mater*. Paucity in LC3-positive astrocytes agrees with previous observations in undamaged spinal cords from rats (see Figure S8 from ref. Liu et al., 2015; and Figure 5 from ref. Hou et al., 2014) as well as in brain sections from transgenic GFP-LC3 mice (Tian et al., 2010; Sarkar et al., 2014). Lack of autophagic structures is coupled to an absence of autophagic initiation in astrocytes, indicated by the absence of Beclin 1 signal reported here and in previous studies (Rami et al., 2008). Therefore, it seems that the autophagic flux remains very low or inactive in spinal cord astrocytes under physiological conditions. Interestingly the only exception, showing high LC3 and Beclin-1 staining, corresponds to the *glia limitans superficialis* (Emsley and Macklis, 2006) that build up the blood brain barrier (BBB) facing the subarachnoid space (Owens et al., 2008). Autophagy may be active in these cells to cope the degradative and recycling requirements of these cells due to their function in the transport of nutrients from the blood and the elimination of noxious metabolites of the CNS (Pardridge, 2007; Abbott et al., 2010).

### SCI induces an accumulation of autophagosomes in neural cells of the spinal cord

Immunoblotting analyses of LC3 and Beclin-1 confirmed that spinal cord injury causes significant changes in autophagy as already described in this and other forms of CNS damage (Kanno et al., 2009a, 2011; Tanabe et al., 2011; Zhang et al., 2013a, 2014; Hou et al., 2014; Liu et al., 2015; Ribas et al., 2015). Increase of LC3-II expression indicates that the abundance of autophagic structures increases at the end of the first week after injury. These results suggest that our mouse model of contusive SCI elicits a delayed autophagic response, as reported before (Kanno et al., 2011; Sekiguchi et al., 2012; Zhang et al., 2013a, 2014; Berliocchi et al., 2015) but contrary to the acute response observed in other studies (Chen et al., 2012; Hao et al., 2013; Hou et al., 2014; Liu et al., 2015; Table 2). Lack of significant changes in the expression levels of Beclin-1 indicates that the increased in autophagic structures is not doe to an increased initiation, contrary to suggestions by several authors (Kanno et al., 2011; Sekiguchi et al., 2012; Wang et al., 2014; Berliocchi et al., 2015), but may result from the impairment of autophagosome degradation (Sarkar et al., 2014; Liu et al., 2015), a possibility supported by the observed accumulation of p62 protein.

**Table 2:** Bibliographical review to autophagic response after SCI.

**Animal/injury/analyses**	**Results**	**References**
Wistar Rats Hemisection at T8 IF of Beclin-1, Atg 5, Atg7 and ULK1 at 30 mpi, 6 hpi, 1, 14, and 42 dpi	IF: increase of initiation markers in damaged axons at 1 dpi; increase of LC3 puncta at 14 dpi	Ribas et al., 2015
Sprague-Dawley (SD) Rats Weight-drop contusion 10 g-25 mm at T9 IB of LC3 at 2, 4 hpi, 1, 3, and 7 dpi IF of LC3 and TEM at 2 hpi	IB: increase of LC3-II at 2 hpi-1 dpi IF/TEM 2 hpi: LC3 and autophagosomes in neurons; absent in astrocytes	Chen et al., 2012
SD Rats 15 g vascular clip compression at T9 IB of LC3 and p62 at 7 dpi IF of LC3 at 7 dpi	IB: increase of LC3-II plus decrease of p62 at 7 dpi IF: increase of LC3 at 7 dpi, no cell type data	Zhang et al., 2013b
SD Rats Weight-drop contusion 10 g-25 mm at T8 IB and IF of LC3, Beclin-1, and p62 at 1, 7, 14, and 35 dpi	IB: Increase of LC3-II and p62 at 1 dpi; no changes in Beclin 1. Blockage of autophagy confirmed by markers of lysosomes IF: blockage in neurons at 1 dpi; in microglia and oligodendrocytes at 1/7 dpi. No changes in astrocytes	Liu et al., 2015
Wistar Rats Weight-drop contusion 10 g-25 mm at T10 qPCR of Beclin-1 and LC3 at 1, 2, 6, 24, 48 and 72 hpi IB and IF of Beclin-1 and LC3 at 2 hpi	qPCR: increase of LC3 and Beclin 1 transcription at 2 hpi IB and IF: increase of protein levels of LC3 and Beclin-1 at 2 hpi	Wang et al., 2015
SD Rats Weight-drop contusion 10 g-25 mm at T10 IB and qPCR of LC3 and Beclin 1 at 1, 2, 6, 24, 48, and 72 hpi IF of LC3 at 2 hpi	qPCR and IB: Increase of protein and mRNA expression of LC3-II y Beclin 1 at 2 hpi IF: increase of LC3 signal in neurons at 2 hpi	Hao et al., 2013
Female SD Rats Weight-drop contusion 10 g-25 mm at T9 IB of LC3 and Beclin 1 at 12 hpi, 1, 3, 7, 14, and 21 dpi	IB: Increase of LC3-II at 3–7 dpi; increase of Beclin-1 at 12–24 hpi	Zhang et al., 2014
C57BL/6J mice Hemisection at T10 IF of LC3 at 4 hpi, 1, 3, 7, and 21 dpi IB of LC3 and TEM at 3 dpi	IF: increase of LC3 staining at 3 dpi among neurons, astrocytes and oligodendrocytes Confirmed by IB and TEM	Kanno et al., 2011
C57BL/6J mice Hemisection at T10 IF of Beclin 1 at 4 hpi, 1, 3, 7, and 21 dpi IB of Beclin 1 and TEM at 3 dpi	IF: increase of Beclin 1 staining at 3 dpi among neurons, astrocytes and oligodendrocytes Confirmed by IB & TEM	Kanno et al., 2009a
White Rabbits Ischemia (15 min in right femoral artery) IB and IHC of LC3 at 8 hpi, 1 and 2 dpi	IB: Peak of LC3-II at 8 hpi IF: LC3 staining in motoneurons at 8 hpi	Baba et al., 2009
SD Rats 30 g force clip compression in T7-T10 IB of Beclin-1, LC3 and p62 at 3, 7, and 14 dpi IF of LC3 + NeuN at 7 dpi	IB: Increase of LC3-II/LC3-I at 7 dpi; Increase of Beclin-1 at 14 dpi; Decrease of p62 at 3, 7 dpi IF: LC3 staining in motoneurons at 7 dpi	Wang et al., 2017
Female C57BL/6J mice Weight-drop contusion 10 g-3 mm at T10 IB of Beclin 1 and LC3 at 1 and 3 dpi IF of Beclin-1 and LC3 at 3 dpi	IB: Increase of LC3-II & Beclin 1 at ¿1 or 3 dpi? IF: increase in the number of LC3 and Beclin-1 positive cells at 3 dpi	Sekiguchi et al., 2012
SD rats Ligation of L5 spinal nerve IF of LC3 and Beclin-1 at 14 dpi	IF: increase of LC3 and Beclin-1 in neurons at 14 dpi; Slight increase in astrocytes, no changes in microglia and oligodendrocytes	Zhang et al., 2013a
SD rats Hemisection between T9/T10 IF of LC3 and Beclin-1 at 4, 8 hpi, 1, 3, 7, and 21 dpi IB of LC3, Beclin-1 and p62 at the same times RT-PCR of Beclin 1 at the same times	IB: increase of LC3 and Beclin-1 peaking at 3 dpi; gradual decrease of p62 RT-PCR: Beclin 1 expression increases from 4–8 hpi and remains overexpressed IF: neurons follow the IB pattern; astrocytes undergo a strong increase at 3 dpi. No data on oligodendrocytes or microglia	Hou et al., 2014
129/Sv mice Complete transection at T9 IB of LC3 and p62 at 1, 3, 7, 14, 21, and 28 dpi IF of LC3 and p62 at 7 dpi	IB: increase of LC3-II y p62 at 7 dpi. Blockage of autophagy IF: Confirmation in motoneurons	Zhang et al., 2017b
C57BL/6J mice Hemisection at T12 IB of Beclin-1 and p62 at 4 dpi	IB: increase of LC3-II at 4 dpi; no changes of Beclin 1 expression	Goldshmit et al., 2015
SD rats Weight-drop contusion of 10 g-30 mm at T9-T10 IB of Beclin 1, LC3 and p62 at 7 dpi	IB: Increase of LC3-II, decrease of p62, and slight increase of Beclin-1 at 7 dpi	Zhao et al., 2017
SD rats 1 min 30 g force clip compression at T9 IB of Beclin 1, LC3 and p62 at 3 dpi IF of LC3 and p62 at 3 dpi	IB: increase of Beclin 1, LC3-II and p62 at 3 dpi IF: increase of LC3 and p62 in motoneurons	Zhang et al., 2017a

*IB, immunoblot; IF, immunofluorescence; TEM, transmission electron microscopy; RT-PCR; RetroTranscription-Polymerase Chain Reaction; mpi, minutes post injury; hpi, hours post injury; dpi, days post injury*.

Histological analyses provide additional information on cell-specific patterns that may help to clarify the actual changes in autophagy after SCI. According to these analyses, autophagic flux undergoes important changes in neurons after SCI. Severed axons are the first neuronal structures to undergo changes. Retraction bulbs increase LC3 and Beclin-1 staining during the first hours after injury, suggesting that both initiation and formation of autophagic vesicles become overactivated after axotomy, as previously described after spinal cord hemisection as well as in an *in vitro* model of axotomy (Bunge, 1973; Ribas et al., 2015). Changes in the neuronal soma become evident later, by the end of the first week after injury, when increased LC3 staining suggests that autophagic structures accumulate in the cell body as previously described (Kanno et al., 2011; Rodríguez-Muela and Boya, 2012; Viscomi et al., 2012), although some studies suggest an earlier onset (Sarkar et al., 2014; Liu et al., 2015). According to the model proposed by Maday an co-workers (Maday and Holzbaur, 2012, 2014, 2016; Maday et al., 2012, 2014), accumulation of autophagic structures at the neuronal soma depends on the balance between the incoming autophagosomes and autolysosomes produced at the axons plus those formed at the soma, and the vesicles being degraded at the soma. Lack of relevant changes in Beclin-1 expression at the soma excludes any critical increase of local initiation. Conversely, the arrival of the autophagic vesicles increasingly formed at the severed axons is a more likely contributor, as demonstrated by Rodriguez-Muela and colleagues in the severed optic nerve (Rodríguez-Muela et al., 2012). Therefore, accumulation of autophagosomes and related structures indicates that their rate of degradation at the soma is not high enough to keep their numbers down to the levels observed before injury. In addition, multiple SCI-driven events such as exposure to glutamate (Kulbe et al., 2014), imbalance of intracellular calcium levels (Engedal et al., 2013), or failure in lysosome activity (Liu et al., 2015) may reduce or even block the capability of neuronal somas to degrade autophagic vesicles and their cargoes as observed in traumatic brain injury (TBI) (Sarkar et al., 2014). The functional consequences of autophagosome accumulation would depend on whether or not this accumulation results from the blockage of the autophagic flux. Blockage would cause autophagic substrates to remain undegraded, leading among others to endoplasmic reticulum stress and ultimately to neuronal death as observed in other models of CNS damage (Sarkar et al., 2014; Liu et al., 2015; Ribas et al., 2015). On the contrary, accumulation of autophagosomes due to enhanced autophagy can be neuroprotective as observed after axotomy of the optic nerve (Rodríguez-Muela et al., 2012), although it may also lead to autophagic death of neurons after SCI (Clark et al., 2008; Sarkar et al., 2014; Liu et al., 2015); however difficulties to define and identify this process (Liu and Levine, 2015) make its participation in SCI highly controversial.

Injury also induces autophagic changes in oligodendrocytes. As in neurons, autophagic structures in these cells increase at the end of the first week after damage, particularly in the regions surrounding the injury site, without signals of increased autophagy initiation (Beclin-1 levels remain unaltered). Previous descriptions in TBI patients and murine models of TBI and SCI (Clark et al., 2008; Sarkar et al., 2014; Liu et al., 2015) suggest that the increase of autophagosome abundance in oligodendrocytes is a general trend after CNS damage. Unfortunately, studies on autophagy in oligodendrocytes are scarce, so we cannot confirm whether the lack of changes in autophagy initiation observed here is also a common trend or just restricted to the present model. Moreover, lack of mechanistic models, such as the one on neuron autophagy proposed by Maday's lab, hinders interpreting the observed changes in autophagy. However, exposure of oligodendrocytes to autophagy-blocking stimuli, such as excitotoxicity and imbalance of intracellular calcium levels (Engedal et al., 2013; Kulbe et al., 2014; Liu et al., 2015), would support the hypothesis that blockage underlies autophagosome accumulation in these cells. Such malfunctioning may be deleterious in SCI conditions, considering the protective role of autophagy in physiological conditions (Mizushima et al., 2008) and its contribution to oligodendrocyte survival and functioning following demyelination in *les* rats (Smith et al., 2013). In fact, oligodendrocyte death after SCI (Lytle and Wrathall, 2007) broadly coincides both in time and location with the accumulation of autophagic vesicles in these cells. However, additional evidences are needed to confirm this relationship and to discard the possibility that the observed increase in autophagosome abundance is indeed a protective response to damage.

Astrocytes in the surroundings of the injury also accumulate autophagic structures at 7 dpi. However, contrary to neurons and oligodendrocytes, astrocytes present a marked staining of Beclin 1 suggesting that autophagy becomes overactivated in these glial cells. Their position at the injury border together with their high GFAP staining suggest that autophagy activation is particularly high in reactive astrocytes. Similar activation has been previously observed in other CNS injury models such as spinal cord hemisection (Kanno et al., 2011), nerve lumbar axotomy (Zhang et al., 2013a) or TBI (Diskin et al., 2005; Erlich et al., 2006; Zhang et al., 2008) although two studies failed to identify it in mouse models of stroke and TBI (Tian et al., 2010; Sarkar et al., 2014). Activation of autophagy is likely a protective mechanism against the deleterious conditions that astrocytes face at the penumbra zone, as occurs when exposed to nutrient deprivation, inflammation, or ethanol (Motori et al., 2013; Korenić et al., 2015). However, Qin et al. (2010) have proposed that increased levels of autophagy in astrocytes may lead to self-digestion and death at least in ischemic brain injury. We cannot reject this possibility, however recent evidences suggest that death of reactive astrocytes after SCI occurs through necroptosis rather than through autophagy (Fan et al., 2016), and that reactive astrocytes showing signs of autophagy activation are TUNEL negative (Diskin et al., 2005; Erlich et al., 2006).

### The complexity of autophagy alterations following SCI: disagreements and consequences for the therapeutic potential of autophagy modulation

Reaching an agreement on how SCI alters the autophagic flux and the pathophysiological consequences of these changes can be key to identify novel therapeutic targets and strategies for SCI treatment. When our observations and those from previous studies on autophagy in SCI (Table 2) are put together, it becomes clear that autophagic activity increases significantly in the damaged spinal cord. However, many relevant aspects of this change strongly differ among studies.

One of these disagreements concerns **the time when autophagic change occurs**. As reflected in Table 2, some references have described a delayed increase of autophagy that reaches its maximum at 3 to 7 days after injury, whereas other observed earlier changes during the first 24 h. Interestingly, all studies employing mouse models only identify delayed changes, whereas early changes predominate in rat models. Therefore, the timing of the autophagic response may add to another neuropathological difference between these two species, such as the cavity formation or scar and inflammatory responses [see Byrnes et al. (2010) and references therein]. However, establishing whether this timing differences are circumstantial or constitute a real neuropathological difference require additional analyses that are beyond the scope of the present study.

Disparities also concern the **cells undergoing autophagic changes**, particularly whether or not autophagy becomes activated in astrocytes after injury. According to Kanno et al. (2009a) and Hou et al. (2014), spinal cord damage activates the autophagic flux (both initiation and vesicle formation) in these glial cells, but neither Liu et al. (2015) nor Chen et al. (2012) could find any evidence to confirm it. According to our analyses, autophagy is inactive in most spinal cord astrocytes. However, it becomes strongly activated among astrocytes surrounding the damaged tissue. Studies in other CNS injuries (Diskin et al., 2005; Erlich et al., 2006; Zhang et al., 2008, 2013a; Kanno et al., 2009a, 2011; Tian et al., 2010; Sarkar et al., 2014) have provided additional evidences that confirm autophagy activation in reactive astrocytes, although a few studies found evidences on the contrary (Sarkar et al., 2014).

A third controversy concerns the **nature of the autophagic changes**. Analyses of LC3 expression by immunoblotting and immunostaining, together with electronic microscopy by different laboratories have led to a general consensus that SCI and related pathologies cause an increase of autophagy -that is, an increase in the abundance of autophagic structures- in the spinal cord (Table 2). There is also an agreement that autophagosomes and related structures increase their abundance in neurons and oligodendrocytes following injury (Table 2), although Zhang and coworkers (Zhang et al., 2013a) failed to observe sign of this increase in oligodendrocytes from spinal cords sampled 14 days after L5 spinal nerve ligation, most likely due to the injury model or the sampling time. Conversely, the causes for such increase of autophagic structures greatly differ among studies, particularly in what concerns to their accumulation in neurons and oligodendrocytes. Immunoblotting evidences of increased levels of Beclin 1 and other markers of autophagy initiation together with a decrease of p62 levels have led several authors to propose that the accumulation of autophagic structures results from the activation of the autophagic flux after the injury (Kanno et al., 2009a, 2011; Viscomi et al., 2012; Zhang et al., 2013b, 2017b; Hou et al., 2014; Wang et al., 2017; Zhao et al., 2017). Immunofluorescence analyses of Beclin 1 have provided further details evidencing the increase of autophagy initiation in neurons and oligodendrocytes of the damaged spinal cord (Kanno et al., 2009a). On the contrary, other studies have observed a constant expression of Beclin 1 and/or increased levels of p62 that suggest that autophagic structures accumulate due to a blockage of the autophagic flux (Liu et al., 2015; Zhang et al., 2017b), with immunofluorescence evidences of such blockage in neurons and oligodendrocytes. Lack of changes in Beclin 1 expression according to our immunoblotting analyses supports a blockage of autophagy in the injured spinal cord. Furthermore, lack of changes in the Beclin 1 signal of neurons and oligodendrocytes revealed by our immunofluorescence analyses indicates that the accumulation of autophagosomes and related structures in the cell body of both cells does not respond to an increase in local initiation. Concomitant accumulation of p62 revealed in our immunoblotting analyses further support a blockage of autophagy that may affect these cells. However, there are alternative interpretations: autophagosomes and related vesicles may accumulate in the soma of neurons and oligodendrocytes due to the increased initiation of autophagy in peripheral compartments (axons in neurons) as we previously discussed, and p62 levels may raise from its overexpression under stress conditions as described previously (Chen et al., 2014; Sahani et al., 2014).

The last and probably the most important controversial aspect of autophagy in the damaged spinal cord concerns the consequences of autophagic changes in the neuropathology of the SCI and how to modulate these changes to improve functional recovery. Autophagy is an essential process for cell survival, whose alterations are commonly associated with pathological conditions, such as neurodegenerative diseases (Shintani and Klionsky, 2004; Rubinsztein, 2006; Mizushima et al., 2008; Settembre et al., 2008b; Nixon, 2013). Almost every article dealing with autophagy after SCI has interpreted the changes they observed from a pathophysiological perspective and proposed consequent detrimental or beneficial effects. However, the heterogeneous responses between and within cell types, or even between cell compartments observed here and in previous studies (Liu et al., 2015) and the strong discrepancies in the changes observed among studies, species, and injury models make establishing autophagic changes as beneficial or detrimental for the damaged spinal cord highly speculative and simplistic in the actual state of knowledge. Pharmacological studies modulating autophagy in the damaged spinal cord can provide additional insights about the contribution of autophagy to the pathophysiology of SCI. Some of these studies have employed rapamycin to promote autophagy in the injured spinal cord leading to lower levels of tissue damage and increased functional recovery (Sekiguchi et al., 2012; Goldshmit et al., 2015). On the basis of these results, autophagy activation after SCI should be considered a beneficial response leading to enhanced cell protection. However, the anti-inflammatory effects of rapamycin, which prevent neuronal cell death (Goldshmit et al., 2015), may also account for the improvements observed after the treatment. Other studies employed alternative drugs such as Simvastatin or pollen typhae (Gao et al., 2015; Wang et al., 2015), however poor characterization of their mechanisms of action prevent determining if modulation of autophagy was the cause or a side effect of the improved spinal cord recovery. In summary, we consider that the evidences on the functional consequences of the autophagic alterations following SCI are far from conclusive. Furthermore, in the light of present and previous results, we consider that we should thoroughly understand the complex and heterogeneous autophagic response triggered by the SCI if we are to develop an autophagy-based therapy with enough guarantee to be tested in human patients.

## Author contributions

TM-G and MN-D conceived, designed the experiments and wrote the manuscript. TM-G, ÁdÁ, MC-L, and IV performed the experiments. TM-G, DR, RM, ÁdÁ, and MN-D performed the analysis. All the authors revised and approved the manuscript.

### Conflict of interest statement

The authors declare that the research was conducted in the absence of any commercial or financial relationships that could be construed as a potential conflict of interest.
