# Supplementary material for: Cell Specific Changes of Autophagy in a Mouse Model of Contusive Spinal Cord Injury
Source: Front Cell Neurosci. 2018 Jun 12;12:164. doi: 10.3389/fncel.2018.00164 (PMC6005838; doi:10.3389/fncel.2018.00164)
Supplement: Supplementary file 1 [file Data_Sheet_1.docx]

Supplementary Material

**CELL SPECIFIC CHANGES OF AUTOPHAGY IN A MOUSE MODEL OF CONTUSIVE SPINAL CORD INJURY**

**Teresa Muñoz-Galdeano, David Reigada, Ángela del Águila, Irene Vélez, Marcos Javier Caballero, Rodrigo M Maza, Manuel Nieto-Diaz***

*** Correspondence:** Manuel Nieto-Diaz: mnietod@sescam.jccm.es

**SUPLEMENTARY FIGURE 1**

**
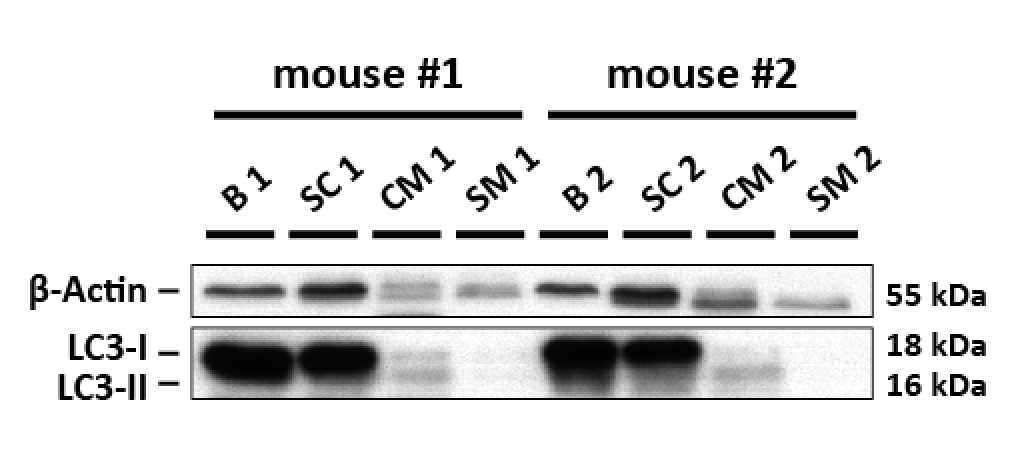
**

**Figure S1. LC3B protein expression in the spinal cord and other tissues from C57BL/6 mice.** Immunoblot of brain (B), spinal cord (SC), cardiac muscle (CM), and striated muscle (SM) tissue samples from undamaged mice. The lipidated (LC3-II, 18kDa) and unlipidated (LC3-I, 16kDa) forms of LC3 can be distinguished in the muscle samples but are obscured by the much more intense LC3-I bands present the brain and spinal cord samples.

**SUPPLEMENTARY FIGURE 2. ImageJ Macro for LC3 quantification in oligodendrocytes**

macro "oligodendrocytes [6]" {

saveAs("Tiff", "raw.tif");

run("Split Channels");

run("Set... ", "zoom=5");

selectWindow("C1-raw.tif");

close();

selectWindow("C3-raw.tif");

run("Set... ", "zoom=5");

run("Z Project...", "start=1 stop=10 projection=[Max Intensity]");

run("Duplicate...", "title=[duplicate]");

selectWindow("duplicate");

run("Set... ", "zoom=5");

selectWindow("C3-raw.tif");

close();

selectWindow("MAX_C3-raw.tif");

run("Duplicate...", "title=[filter]");

selectWindow("filter");

run("Median...", "radius=20");

selectWindow("MAX_C3-raw.tif");

setTool("polygon");

print("Nuclei image preprocessed");

waitForUser ("User input", "Select spinal cord area and press OK);

selectWindow("MAX_C3-raw.tif");

run("Median...", "radius=2");

run("Subtract...", "value=25");

run("Multiply...", "value=2");

run("Make Inverse");

run("Clear", "slice");

run("Invert");

imageCalculator("Subtract create", "MAX_C3-raw.tif","filter");

print("Result of MAX_C3-raw.tif");

selectWindow("Result of MAX_C3-raw.tif");

run("Make Binary");

selectWindow("Result of MAX_C3-raw.tif");

setTool("polygon");

print("Nuclei image preprocessed");

waitForUser ("User input", "Delete artifacts and press OK");

run("Analyze Particles...", "size=15-infinite circularity=0.00-1.00 show=Masks clear add");

print("Mask of Result of MAX_C3-raw.tif");

print("binarized image");

selectWindow("C2-raw.tif");

run("Set... ", "zoom=5");

run("Z Project...", "start=1 stop=10 projection=[Max Intensity]");

roiManager("Show all");

roiManager("Measure");

selectWindow("C2-raw.tif");

close();

selectWindow("filtro");

close();

**SUPPLEMENTARY FIGURE 3. ImageJ Macro for LC3 quantification in neurons**

macro "neurons [6]" {

saveAs("Tiff", "raw.tif");

run("Split Channels");

run("Set... ", "zoom=5");

selectWindow("C1-raw.tif");

run("Set... ", "zoom=5");

run("Z Project...", "start=1 stop=10 projection=[Max Intensity]");

selectWindow("C1-raw.tif");

close();

selectWindow("MAX_C1-raw.tif");

run("Median...", "radius=2");

saveAs("Tiff", "NUnn.tif");

run("Set... ", "zoom=5");

run("Duplicate...", "title=NUt.tif");

run("Set... ", "zoom=5");

print ("projecting focal planes of NeuN");

selectWindow("C2-raw.tif");

run("Z Project...", "start=1 stop=5 projection=[Max Intensity]");

selectWindow("C2-raw.tif");

close();

print("done");

print("preprocessing NeuN image");

selectWindow("MAX_C2-raw.tif");

run("Original Scale");

run("Median...", "radius=2");

run("Subtract Background...", "rolling=30");

run("Brightness/Contrast...");

setTool("polygon");

print("NeuN image preprocessed");

waitForUser ("User input", "Select the gray substance and press OK");

run("Create Mask");

saveAs("Tiff", "Graymatter.tif");

run("Set... ", "zoom=5");

selectWindow("MAX_C2-raw.tif");

run("Select None");

run("Duplicate...", "title=cord.tif");

run("Set... ", "zoom=5");

selectWindow("MAX_C2-raw.tif");

run("Auto Threshold", "method=[Try all] white");

Dialog.create("User input");

Dialog.addMessage("choose the most apropriate threshold\n and apply it at image/adjust/autothreshold\n close Montage window\n once thresholded press 7")

Dialog.show();

selectWindow("C2-raw.tif");

run("Set... ", "zoom=5");

run("Z Project...", "start=1 stop=10 projection=[Max Intensity]");

roiManager("Show all");

roiManager("Measure");

selectWindow("C2-raw.tif");

close();

**SUPLEMENTARY FIGURE 4**

data<-read.table(“data.txt", header=TRUE)
res<-lm(LC3~BECLIN, data=data)
summary(res)
res2<-lm(LC3~BECLIN*INJURY, data=data)
summary(res2)
anova(res, res2)

**Figure S4.** R Script employed to carry out lineal regression in the analysis of the relationship between LC3 and Beclin 1 expression and the effect of the injury.

**SUPLEMENTARY FIGURE 5**

**A**


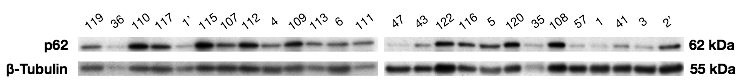


**B**

|  | **Control** | **2 hpi** | **1 dpi** | **3 dpi** | **7 dpi** | **14 dpi** |
| --- | --- | --- | --- | --- | --- | --- |
| **# mouse** | 1, 3, 4, 35, 36, and 113 | 1’, 2’, 5, 6, and 116 | 47, 57, 117, and 119 | 41, 43, and 120 | 110, 111, and 112 | 107, 108, 109, and 122 |

**Figure S5.** **p62 accumulates after SCI.** **(A)** Immunoblot of SQSTM1/p62 in spinal cord lysates from control and injured animals. Samples were randomly loaded onto two gels to ensure reliability and avoid biases between comparisons. **(B)** Mouse codes for each post-injury time point.

**SUPLEMENTARY FIGURE 6**

**
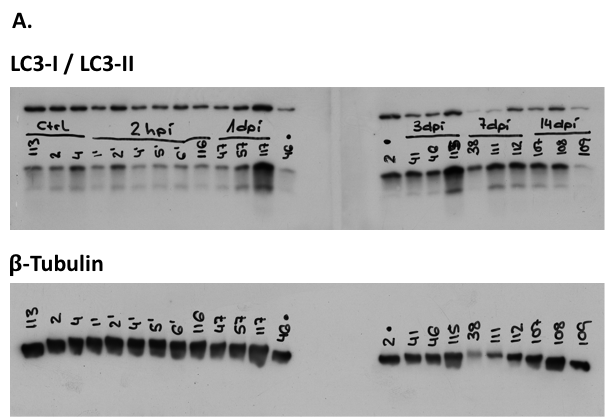
**

**
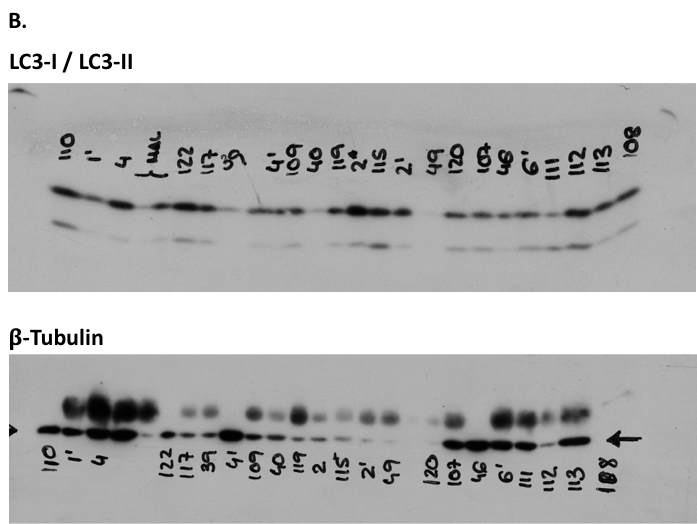
**

|  | **Control** | **2 hpi** | **1 dpi** | **3 dpi** | **7 dpi** | **14 dpi** |
| --- | --- | --- | --- | --- | --- | --- |
| **# mouse** | 2, 4 and 113 | 1’, 2’, 4, 5, 6’, and 116 | 47, 49, 57, 117, and 119 | 41, 46 and 120 | 38, 39, 40, 110, 111, and 112 | 107, 108, 109, and 122 |

**Figure S6.** Unprocessed scan of the LC3 immunoblots employed for illustration (**A**) and quantification (**B**) in figure 2. Samples 2 and 46 in **A** were replicated in both membranes for comparison. Conditions of the samples included in B are detailed in the table.

**SUPLEMENTARY FIGURE 7**

**
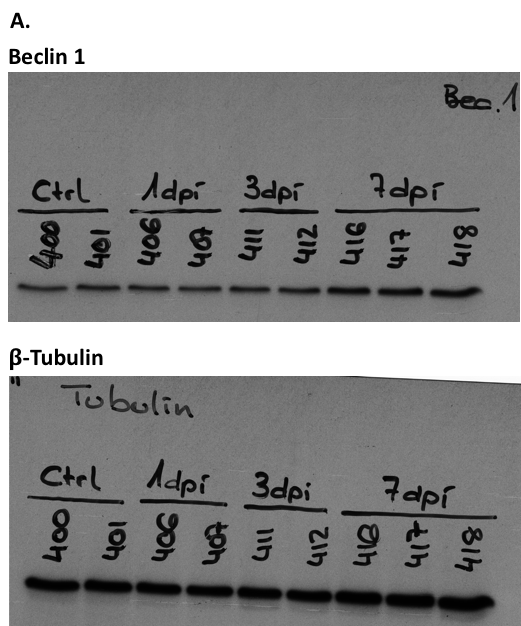
**

**
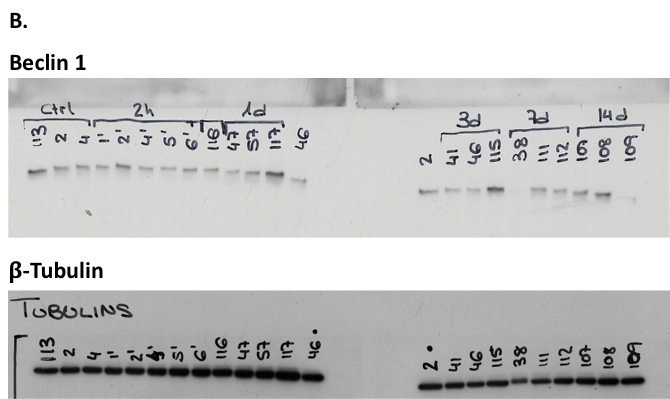
**

**Figure S7.** Unprocessed scan of the Beclin-1 immunoblots employed for illustration (**A**) and for quantification (**B**) in figure 9.
